# Greater daily glucose variability and lower time in range assessed with continuous glucose monitoring are associated with greater aortic stiffness: The Maastricht Study

**DOI:** 10.1007/s00125-021-05474-8

**Published:** 2021-05-15

**Authors:** Yuri D. Foreman, William P. T. M. van Doorn, Nicolaas C. Schaper, Marleen M. J. van Greevenbroek, Carla J. H. van der Kallen, Ronald M. A. Henry, Annemarie Koster, Simone J. P. M. Eussen, Anke Wesselius, Koen D. Reesink, Miranda T. Schram, Pieter C. Dagnelie, Abraham A. Kroon, Martijn C. G. J. Brouwers, Coen D. A. Stehouwer

**Affiliations:** 1 CARIM School for Cardiovascular Diseases, Maastricht University, Maastricht, the Netherlands; 2 Department of Internal Medicine, Maastricht University Medical Centre+, Maastricht, the Netherlands; 3 Department of Clinical Chemistry, Central Diagnostic Laboratory, Maastricht University Medical Centre+, Maastricht, the Netherlands; 4 Department of Internal Medicine, Division of Endocrinology and Metabolic Disease, Maastricht University Medical Centre+, Maastricht, the Netherlands; 5 CAPHRI Care and Public Health Research Institute, Maastricht University, Maastricht, the Netherlands; 6 Heart and Vascular Center, Maastricht University Medical Centre+, Maastricht, the Netherlands; 7 Department of Social Medicine, Maastricht University, Maastricht, the Netherlands; 8 Department of Epidemiology, Maastricht University, Maastricht, the Netherlands; 9 NUTRIM School for Nutrition and Translational Research in Metabolism, Department of Complex Genetics and Epidemiology, Maastricht University, Maastricht, the Netherlands; 10 Department of Biomedical Engineering, Maastricht University, Maastricht, the Netherlands

**Keywords:** Arterial stiffness, Continuous glucose monitoring, Glucose variability, Time in range

## Abstract

**Aims:**

CVD is the main cause of morbidity and mortality in individuals with diabetes. It is currently unclear whether daily glucose variability contributes to CVD. Therefore, we investigated whether glucose variability is associated with arterial measures that are considered important in CVD pathogenesis.

**Methods:**

We included participants of The Maastricht Study, an observational population-based cohort, who underwent at least 48 h of continuous glucose monitoring (CGM) (*n* = 853; age: 59.9 ± 8.6 years; 49% women, 23% type 2 diabetes). We studied the cross-sectional associations of two glucose variability indices (CGM-assessed SD [SD_CGM_] and CGM-assessed CV [CV_CGM_]) and time in range (TIR_CGM_) with carotid–femoral pulse wave velocity (cf-PWV), carotid distensibility coefficient, carotid intima–media thickness, ankle–brachial index and circumferential wall stress via multiple linear regression.

**Results:**

Higher SD_CGM_ was associated with higher cf-PWV after adjusting for demographics, cardiovascular risk factors and lifestyle factors (regression coefficient [B] per 1 mmol/l SD_CGM_ [and corresponding 95% CI]: 0.413 m/s [0.147, 0.679], *p* = 0.002). In the model additionally adjusted for CGM-assessed mean sensor glucose (MSG_CGM_), SD_CGM_ and MSG_CGM_ contributed similarly to cf-PWV (respective standardised regression coefficients [st.βs] and 95% CIs of 0.065 [−0.018, 0.167], *p* = 0.160; and 0.059 [−0.043, 0.164], *p* = 0.272). In the fully adjusted models, both higher CV_CGM_ (B [95% CI] per 10% CV_CGM_: 0.303 m/s [0.046, 0.559], *p* = 0.021) and lower TIR_CGM_ (B [95% CI] per 10% TIR_CGM_: −0.145 m/s [−0.252, −0.038] *p* = 0.008) were statistically significantly associated with higher cf-PWV. Such consistent associations were not observed for the other arterial measures.

**Conclusions:**

Our findings show that greater daily glucose variability and lower TIR_CGM_ are associated with greater aortic stiffness (cf-PWV) but not with other arterial measures. If corroborated in prospective studies, these results support the development of therapeutic agents that target both daily glucose variability and TIR_CGM_ to prevent CVD.

**Graphical abstract:**

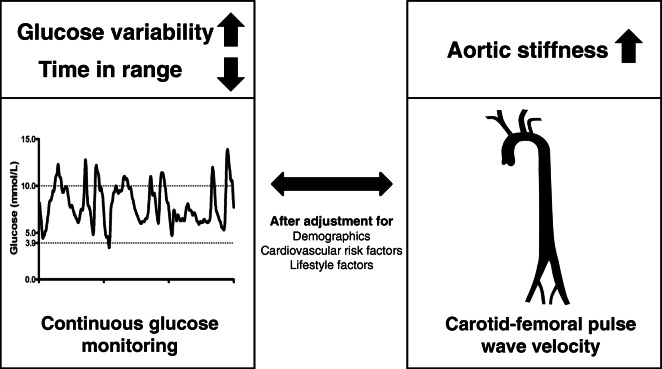

**Supplementary Information:**

The online version contains supplementary material available at 10.1007/s00125-021-05474-8.

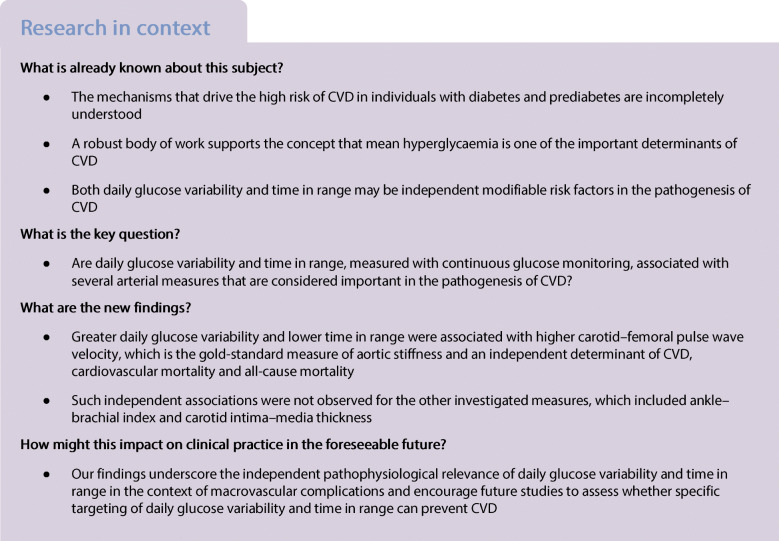



## Introduction

CVD is the main cause of morbidity and mortality in individuals with type 2 diabetes [1]. Additionally, individuals with prediabetes are already at an elevated risk of CVD [2]. Hyperglycaemia contributes to this CVD risk, in part, by its adverse effects on arterial stiffness [3–5], atherosclerosis [1, 6], and large-artery endothelial function [5, 7]. Accordingly, both achieving and maintaining normoglycaemia are important for reducing CVD risk [1]. However, current treatment modalities have not been able to fully normalise the elevated CVD risk of individuals with type 2 diabetes [1]. A better understanding of the involved pathophysiologic processes could yield new therapeutic targets to further reduce CVD risk.

Glucose variability (GV) is thought to contribute to the development of CVD, irrespective of mean glucose values. Notably, two types of GV need to be distinguished, as they are measured differently and represent different underlying aetiologic concepts [8, 9]. Short-term (or daily) GV reflects actual glucose fluctuations over the day [9, 10]. By contrast, long-term (or visit-to-visit) GV reflects variance in classic glycaemic indices (e.g., HbA_1c_) that have been periodically measured over weeks, months, or years [8, 9]. While long-term GV may assess daily glucose fluctuations to some extent, it is viewed to largely represent difficult to measure factors that affect glycaemic control (e.g., therapy adherence, multimorbidity, infections) [8]. Whereas multiple studies have shown that long-term GV is independently associated with CVD and all-cause mortality [8, 11–15], the association between daily GV and CVD has only been sparsely investigated [16].

In general, the study of incident CVD requires both a substantial sample size and an ample follow-up period. Large-scale measurement of daily GV with the gold-standard method (i.e., continuous glucose monitoring [CGM]) [17] has been challenging until recently, in part because of costliness and relative invasiveness [18]. Therefore, most studies on this topic have cross-sectionally associated daily GV with measures that reflect the aforementioned processes leading to CVD [19–25]. Importantly, however, these studies either did not adjust for certain important potential confounders [20–23] or assessed daily GV with less precise methods than CGM [24, 25].

Hence, we investigated whether daily GV is associated with arterial measures that are considered important in CVD pathogenesis in a population-based cohort study. We studied whether the associations were independent of key demographics, cardiovascular risk factors and lifestyle factors, and assessed to what extent the associations were explained by mean glycaemia. Based on previous work [25], we hypothesised that CGM-derived indices of GV would be most strongly associated with carotid–femoral pulse wave velocity (cf-PWV), which is the gold-standard measure of aortic stiffness because of its independent association with incident CVD, cardiovascular mortality and all-cause mortality [26–28]. In secondary analyses, we assessed the associations of CV (CV_CGM_), an index that is intrinsically adjusted for mean glycaemia, and time in range (TIR_CGM_), an emerging glycaemic index that is partly determined by GV [29], with the same arterial outcome variables.

## Methods

### Study population and design

We used data from The Maastricht Study, an observational, prospective, population-based cohort study. The rationale and methodology have been described previously [30]. In brief, The Maastricht Study focuses on the aetiology, pathophysiology, complications, and comorbidities of type 2 diabetes, and is characterised by an extensive phenotyping approach. All individuals aged between 40 and 75 years and living in the southern part of the Netherlands were eligible for participation. Participants were recruited through mass media campaigns and from the municipal registries and the regional Diabetes Patient Registry via mailings. For reasons of efficiency, recruitment was stratified according to known type 2 diabetes status, with an oversampling of individuals with type 2 diabetes. In general, the examinations of each participant were performed within a time window of 3 months. The Maastricht Study has been approved by the institutional medical ethical committee (NL31329.068.10) and the Minister of Health, Welfare and Sports of the Netherlands (Permit 131088-105234-PG). All participants gave written informed consent.

### Continuous glucose monitoring

The rationale and methodology of CGM (iPro2 and Enlite Glucose Sensor; Medtronic, Tolochenaz, Switzerland) have been described previously [31]. From 19 September 2016 to 13 September 2018, all participants were invited to undergo CGM as part of their regular work-up at The Maastricht Study. To accelerate the inclusion process and to ensure inclusion of a sufficient number of participants with prediabetes and type 2 diabetes, we re-invited a selected group of participants who had recently visited The Maastricht Study to undergo CGM as a separate research visit (further referred to as ‘catch-up visit’). The CGM device was worn on the lower abdomen and recorded subcutaneous interstitial glucose values (range: 2.2–22.2 mmol/l) every 5 min for a 7-day period. Participants were asked to self-measure their blood glucose four times daily (Contour Next; Ascensia Diabetes Care, Mijdrecht, the Netherlands) for retrospective CGM calibration. Participants were blinded to the CGM recording, but not to the self-measured values. Diabetes medication use was allowed, and no instructions on diet or physical activity were given.

The first 24 h of CGM were excluded because of insufficient calibration. Next, we excluded individuals with less than 24 h of recording (less than one data day). Then, we calculated per participant mean sensor glucose (MSG_CGM_), SD_CGM_, CV_CGM_ (i.e., SD_CGM_/MSG_CGM_ × 100%) and TIR_CGM_ (i.e., % of time between 3.9 and 10.0 mmol/l) using the total recording period. Based on international consensus, we used SD_CGM_ and CV_CGM_ as indices of GV [17].

### Arterial measurements

The rationale and methodology of the arterial measurements have been described previously [25, 32, 33]. We assessed cf-PWV using applanation tonometry (SphygmoCor, Atcor Medical, Sydney, Australia) [26] and used the median of at least three consecutive cf-PWV recordings in our analyses. Because of its established clinical relevance [26–28], cf-PWV was our main outcome measure of interest.

In addition, we measured the left common carotid artery with the use of an ultrasound scanner equipped with a 7.5 MHz linear probe (MyLab 70, Esaote Europe, Maastricht, the Netherlands) to assess local carotid distension, intima–media thickness (cIMT), and interadventitial diameter (IAD) [34]. We quantified local arterial stiffness by calculating the carotid distensibility coefficient (carDC) according to the following formula: carDC = (2 × ΔD × IAD + ΔD^2^)/(braPP×IAD^2^), where ΔD = distension and braPP = brachial pulse pressure [35]. We defined cIMT as the distance between the lumen–intima and media–adventitia interfaces of the far (posterior) wall [34], and IAD as the distance between the media–adventitia interfaces of the near and far wall. The median carDC, cIMT and IAD of three consecutive measurements were used.

We calculated carotid lumen diameter (LD) according to the following formula [36]: LD = IAD – (2 × cIMT). In parallel with the vascular measurements, we also determined mean heart rate and mean arterial pressure (MAP) every 5 min with an oscillometric device (Accutorr Plus, Datascope, Montvale, NJ, USA). We calculated mean circumferential wall stress (CWS_mean_) and pulsatile circumferential wall stress (CWS_puls_) using the Lamé equation as follows: CWS_mean_ = [MAP×(LD/2)]/cIMT and CWS_puls_ = [braPP×(LD/2)]/cIMT [32].

Last, the Omron VP2000 (Omron, Kyoto, Japan) was used to automatically determine the ankle–brachial index (ABI) based on simultaneous BP measurements at both ankles and upper arms. The left and right ABI were calculated by dividing the systolic BP measured at the ankle by the highest systolic BP measured at either upper arm. We used the lowest ABI in our analyses and excluded individuals with an ABI above 1.4 [37].

### Measurement of covariates

As described previously [30], we categorised glucose metabolism status (GMS) based on a standardised 2 h 75 g OGTT and the participant’s medication use as either normal glucose metabolism (NGM), prediabetes, or type 2 diabetes [38]. Participants who used insulin or had a fasting plasma glucose value above 11.0 mmol/l did not undergo the OGTT. In addition, we assessed educational level (low, intermediate, high), moderate-to-vigorous physical activity, smoking status (never, former, current), alcohol use (none, low, high), and history of CVD by questionnaire. We also calculated the Dutch Healthy Diet index sum score, a measure of adherence to the Dutch dietary guidelines 2015 [39] based on a food frequency questionnaire [40]; assessed lipid-modifying, antihypertensive and glucose-lowering medication use as part of a medication interview; measured weight, height and waist circumference during a physical examination; calculated BMI; measured office and 24 h ambulatory BP; measured HbA_1c_ and lipid profile in fasting venous blood samples; measured albumin excretion in two 24 h urine collections; and calculated the eGFR based on serum creatinine only, as cystatin C values were not presently available in this subpopulation.

### Statistical analysis

Normally distributed data are presented as mean and SD, non-normally distributed data as median and IQR, and categorical data as *n* (%). We used multiple linear regression with a complete-case approach to study the associations of daily GV with arterial measures. The crude analyses only included SD_CGM_ as a determinant. Model 1 was adjusted for demographics: age, sex and education level. Model 2 was additionally adjusted for cardiovascular risk and lifestyle factors: MAP (in case of cf-PWV, carDC, and CWS_puls_), office systolic BP (in case of cIMT and ABI), braPP (in case of CWS_mean_), mean heart rate (in case of cf-PWV and ABI only), BMI, total-to-HDL-cholesterol levels, smoking status, alcohol use and antihypertensive and lipid-modifying drug use. To study its contribution relative to SD_CGM_, the associations were further adjusted for MSG_CGM_ in an additional model (i.e., model 2 + MSG_CGM_). The main regression results are presented as regression coefficients (B) with corresponding 95% CI and *p* values.

We presumed the reliability of our model 2 + MSG_CGM_ results to be negatively impacted by multicollinearity because of the strong correlation between SD_CGM_ and MSG_CGM_ (rho = 0.69) [41]. Hence, we additionally performed ridge regression, an L2-regularised form of linear regression (formula provided in the electronic supplementary material [ESM] Methods), which is a valid statistical method to counter a degree of model instability caused by multicollinearity [42]. Ridge regression estimates are computed according to the combination of the residual sum of squares, characteristic of regular linear regression, and predefined penalisation of the coefficients. As such, it slightly biases the regression coefficients and can strongly reduce inflated variances that arise when high levels of multicollinearity are present. We pragmatically chose the level of penalisation based on the lambda (λ) required to reduce the variance inflation factor (VIF) of model 2 + MSG_CGM_ back to the VIF of model 2 (or halfway back). The ridge regression results are presented as standardised regression coefficients (st.β) with 95% CIs and *p* values. The median st.βs (95% CIs) were estimated with use of resampling (1000 bootstrap).

In secondary analyses, we replaced the main determinant SD_CGM_ with CV_CGM_ and TIR_CGM_. For clarity, the regression coefficients of both indices are presented per 10% difference instead of per 1%. To further explore the clinical applicability of our results in the context of the International Consensus on TIR_CGM_ [43], we repeated the analyses with TIR_CGM_ ≥ 70% (yes/no) as the main determinant. In addition, we investigated whether the associations were modified by sex [44], age [25], or (type 2) diabetes status by adding interaction terms (e.g., SD_CGM_ × sex) to model 2.

To test the robustness of our main findings, we performed several sensitivity analyses by (1) replacing MSG_CGM_ with GMS, HbA_1c_ or fasting plasma glucose; (2) adding physical activity and diet as a separate model because many missing values were observed for these confounders (ESM Table 1); (3) adding specific variables (eGFR, urinary albumin excretion, history of CVD) as a separate model since they may introduce overadjustment bias [45]; (4) substituting office systolic BP with ambulatory systolic BP; and (5) excluding individuals with type 1 diabetes, individuals with CGM data gaps, individuals who underwent CGM as part of a ‘catch-up visit’, or individuals with a suboptimal CGM recording period (i.e., less than two data days) [31]. Last, we also repeated the primary analyses with MSG_CGM_ as the main determinant.

We considered a *p* value of <0.05 statistically significant. Statistical analyses were performed with use of the Statistical Package for Social Sciences (version 25.0; IBM, Chicago, Illinois, USA) and the R programming language (version 3.6.1; R Foundation for Statistical Computing, Vienna, Austria) with package glmnet (version 4.0.2).

## Results

### Study population characteristics

The total CGM study population comprised 853 individuals (age: 59.9 ± 8.6 years; 49% women, 23% type 2 diabetes). Because outcome and covariate data could not be obtained in all individuals (ESM Fig. 1, ESM Table 1), the number of participants who were included in the different regression analyses varied (*n* = 643–816). Table 1 shows the participant characteristics of the largest sample size (i.e., ABI study population) stratified according to tertiles of SD_CGM_. With higher GV, participants were older, more often male, and were generally characterised by a more unfavourable cardiometabolic profile (i.e., higher HbA_1c_, BP and BMI values and more often current smoker). GMS did not fully correspond with daily GV. Namely, 31 (17%) of the 185 individuals with type 2 diabetes were not in the highest tertile of SD_CGM_, participants with prediabetes were evenly distributed between the tertiles, and 58 (13%) of the 454 individuals with NGM were not in the lowest or middle tertiles. ESM Table 2 and ESM Figs 2–4 additionally show that the different GMS categories have substantially overlapping SD_CGM_ values.

**Table 1:** Characteristics of ABI study population (*n* = 816) stratified according to tertiles of SD_CGM_

Characteristic	First SD_CGM_ tertile: 0.32–0.72 mmol/l (*n* = 276)	Second SD_CGM_ tertile: 0.73–1.00 mmol/l (*n* = 267)	Third SD_CGM_ tertile: 1.01–4.81 mmol/l (*n* = 273)
Demographics			
Age, years	57.8 ± 8.9	59.3 ± 8.7	62.1 ± 7.7
Women, *n*	147 (53.3)	126 (47.2)	125 (45.8)
Education (low/medium/high)			
*n*	63/76/137	86/80/101	107/71/95
%	22.8/27.5/49.6	32.2/30.0/37.8	39.2/26.0/34.8
Glycaemic variables			
GMS, NGM/PreD/T2D/T1D			
*n*	230/40/6/0	166/76/25/0	58/59/154/2
%	83.3/14.5/2.2/0	62.2/28.5/9.4/0	21.2/21.6/56.4/0.7
Newly diagnosed T2D	6 (2.2)	18 (6.7)	44 (16.1)
FPG, mmol/l	5.1 [4.9–5.5]	5.4 [5.0–5.9]	6.5 [5.4–7.6]
2 h post-load glucose, mmol/l	5.5 [4.7–6.9]	6.4 [5.2–8.0]	10.3 [7.2–14.5]
MSG_CGM_, mmol/l	5.7 [5.4–6.0]	6.0 [5.7–6.3]	7.1 [6.4–8.1]
SD_CGM_, mmol/l	0.63 [0.55–0.68]	0.84 [0.77–0.93]	1.40 [1.17–1.86]
CV_CGM_, %	10.8 [9.9–11.7]	14.0 [13.0–15.3]	19.9 [17.5–23.9]
TIR_CGM_, %	100.0 [100.0–100.0]	100.0 [99.5–100.0]	94.6 [82.1–98.4]
HbA_1c_			
%	5.4 [5.2–5.5]	5.5 [5.4–5.7]	6.0 [5.6–6.8]
mmol/mol	35.0 [33.0–37.0]	37.0 [35.0–39.0]	42.0 [38.0–51.0]
Diabetes medication use, n	0 (0)	6 (2.2)	96 (35.2)
Insulin	0 (0)	1 (0.4)	19 (7.0)
Metformin	0 (0)	6 (2.2)	91 (33.3)
Sulfonylureas	0 (0)	0 (0)	21 (7.7)
GLP-1 analogues	0 (0)	0 (0)	4 (1.5)
DDP-4 inhibitors	0 (0)	0 (0)	1 (0.4)
SGLT-2 inhibitors	0 (0)	0 (0)	1 (0.4)
Lifestyle factors			
BMI, kg/m^2^	26.1 ± 3.7	26.7 ± 3.9	28.3 ± 4.8
Waist circumference, cm			
Men	98.8 ± 9.9	100.7 ± 10.6	106.3 ± 12.4
Women	87.2 ± 10.7	90.4 ± 11.5	94.2 ± 12.8
Physical activity, h/week	12.5 [7.8–18.5]	12.5 [7.5–19.6]	11.5 [6.8–17.9]
Dutch healthy diet index, (range: 0–150)	85.4 ± 17.3	84.5 ± 16.2	81.3 ± 14.6
Alcohol use (none/low/high)			
*n*	38/179/59	36/180/51	69/164/40
%	13.8/64.9/21.4	13.5/67.4/19.1	25.3/60.1/14.7
Smoking (never/former/current)			
*n*	122/126/28	100/135/32	95/136/42
%	44.2/45.7/10.1	37.5/50.6/12.0	34.8/49.8/15.4
Cardiovascular risk factors			
History of CVD	41 (14.9)	28 (10.6)	53 (19.4)
Office systolic BP, mmHg	129.0 ± 17.5	133.3 ± 17.9	137.0 ± 17.9
Office diastolic BP, mmHg	73.7 ± 9.8	75.4 ± 10.4	75.9 ± 10.2
MAP, mmHg	95.5 ± 10.9	96.8 ± 10.7	98.6 ± 10.7
Mean heart rate, beats/min	59.2 ± 8.1	60.3 ± 8.6	63.3 ± 8.9
Antihypertensive medication use, n	58 (21.0)	84 (31.5)	142 (52.0)
Total-to-HDL-cholesterol ratio	3.3 [2.8–4.3]	3.6 [2.9–4.3]	3.6 [2.8–4.3]
Triacylglycerols, mmol/l	1.2 [0.9–1.5]	1.3 [0.9–1.7]	1.4 [1.0–1.9]
Lipid-modifying medication use, n	31 (11.2)	42 (15.7)	128 (46.9)
eGFR, ml min^−1^ [1.73 m]^−2^	81.8 ± 13.0	79.8 ± 13.8	80.0 ± 10.2
Albuminuria, n	7 (2.5)	23 (8.6)	33 (12.2)
Outcome measures			
cf-PWV, m/s	8.3 ± 1.8	8.5 ± 1.9	9.5 ± 2.5
carDC 10^−3^/kPa	16.3 ± 5.8	16.5 ± 5.9	14.9 ± 6.1
cIMT, μm	865.6 ± 144.0	899.2 ± 152.3	906.7 ± 160.2
ABI	1.14 ± 0.10	1.14 ± 0.10	1.13 ± 0.12
ABI < 0.9, *n*	6 (2.2)	8 (3.0)	10 (3.7)
CWS_mean_, kPa	43.8 [38.1–49.5]	44.0 [37.7–49.7]	44.3 [37.9–52.1]
CWS_puls_, kPa	21.7 [18.6–26.1]	22.5 [18.7–26.5]	23.2 [19.7–29.1]

Data are reported as mean ± SD, median [IQR], or number (%) as appropriate

Data represent the study population of participants with complete data on determinant, outcome (i.e., ABI) and confounders

PreD, prediabetes; T2D, type 2 diabetes; T1D, type 1 diabetes, GLP-1 glucagon-like peptide-1; DPP-4 dipeptidase-4; SGLT2, sodium−glucose cotransporter 2

### Daily GV and arterial stiffness

**Fig. 1:**
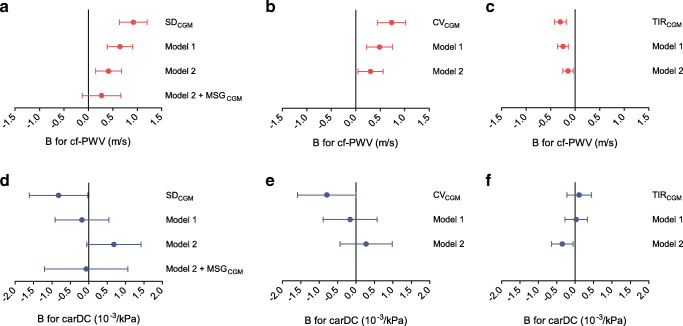
Multivariable-adjusted associations of SD_CGM_, CV_CGM_ and TIR_CGM_ with measures of arterial stiffness. Regression coefficients (B) indicate the mean difference (95% CI) associated with 1 mmol/l increase in SD_CGM_ or 10% increase in CV_CGM_ or TIR_CGM_. (**a**–**c**) Associations with cf-PWV and (**d**–**f**) associations with carDC. Model 1: adjusted for age, sex and education. Model 2: additionally adjusted for MAP, mean heart rate (in the case of cf-PWV only), BMI, smoking status, alcohol use, total-to-HDL-cholesterol levels and use of antihypertensive and lipid-modifying drugs. Model 2 + MSG_CGM_: additionally adjusted for mean sensor glucose

Figure 1 and ESM Table 3 show the associations of SD_CGM_ with cf-PWV and carDC estimated by use of multiple linear regression. Higher SD_CGM_ was statistically significantly associated with higher cf-PWV after adjustment for demographics, cardiovascular risk factors and lifestyle factors (model 2, B: 0.413 m/s [0.147, 0.679], *p* = 0.003). Although numerically, the regression estimate was attenuated by a third after additional adjustment for MSG_CGM_ (model 2 + MSG_CGM_, B: 0.270 m/s [−0.125, 0.666], *p* = 0.180), the coefficients were not statistically significantly different.

Table 2 shows the fully adjusted st.βs of SD_CGM_ and MSG_CGM_, as estimated with ridge regression, to allow better comparison of the strength of association of both indices with cf-PWV. The coefficients were comparable and both not statistically significant (st.β: 0.065 [−0.018, 0.167], *p* = 0.160 for SD_CGM_; and st.β: 0.059 [−0.043, 0.164], *p* = 0.272 for MSG_CGM_).

**Table 2:** Standardised regression coefficients of SD and mean sensor glucose in the fully adjusted models with arterial outcome variables

Arterial outcome variable	Ridge regression penalisation (λ)	SD_CGM_ (st.β, 95% CI)	*p* value	MSG_CGM_ (st.β, 95% CI)	*p* value
cf-PWV, SD (n = 643)	λ = 0.11	0.065 (−0.018, 0.167)	0.160	0.059 (−0.043, 0.164)	0.272
carDC, SD (*n* = 725)	λ = 0.12	−0.003 (−0.097, 0.092)	0.952	0.088 (−0.014, 0.184)	0.102
cIMT, SD (*n* = 726)	λ = 0.12	−0.007 (−0.123, 0.111)	0.916	0.078 (−0.038, 0.207)	0.198
ABI, SD (n = 816)	λ = 0.11	−0.033 (−0.071, 0.002)	0.060	−0.008 (−0.032, 0.017)	0.548
CWS_mean_, SD (n = 725)	λ = 0.12	−0.059 (−0.169, 0.066)	0.318	0.082 (−0.044, 0.204)	0.180
CWS_puls_, SD (n = 725)	λ = 0.12	−0.045 (−0.145, 0.053)	0.374	0.042 (−0.055, 0.138)	0.410

Associations were adjusted for age, sex, educational level, BMI, smoking status, alcohol use, total-to-HDL-cholesterol levels, use of antihypertensive and lipid-modifying drugs, and the other CGM-assessed index. Further, cf-PWV was additionally adjusted for MAP and heart rate; carDC and CWS_puls_ were additionally adjusted for MAP; cIMT was additionally adjusted for office systolic BP; ABI was additionally adjusted for office systolic BP and heart rate; and CWS_mean_ was additionally adjusted for brachial pulse pressure. All coefficients were estimated by use of ridge regression. Point estimates and 95% CIs were calculated by use of 1000 bootstrap estimates

Standardised regression coefficients (st.β) indicate the median difference (95% CI) associated with 1 SD higher SD_CGM_ or MSG_CGM_

In the cf-PWV study population, 1 SD corresponds to 0.57 mmol/l for SD_CGM_, 1.3 mmol/l for MSG_CGM_, and 2.2 m/s for cf-PWV. In the carDC, cIMT, and CWS study populations, 1 SD corresponds to 0.57 mmol/l for SD_CGM_, 1.3 mmol/l for MSG_CGM_, 6.0 10^−3^/kPa for carDC, 152.7 μm for cIMT, 10.2 kPa for CWS_mean_, and 6.6 kPa for CWS_puls_. In the ABI study population, 1 SD corresponds to 0.56 mmol/l for SD_CGM_, 1.3 mmol/l for MSG_CGM_, and 0.11 for ABI

In the analysis with CV_CGM_ as the determinant, the association with cf-PWV was statistically significant after full adjustment (model 2, B per 10% CV_CGM_: 0.303 m/s [0.046, 0.559], *p* = 0.021; ESM Table 4). In line with the main results, higher TIR_CGM_ was independently associated with lower cf-PWV (model 2, B per 10% TIR_CGM_: −0.145 m/s [−0.252, −0.038] *p* = 0.008; Fig. 1, ESM Table 5). Correspondingly, TIR_CGM_ ≥ 70% was independently associated with lower cf-PWV (model 2, B: −1.098 m/s [−1.745, −0.451], *p* = 0.001; ESM Table 6).

SD_CGM_ was not associated with carDC after adjustment for demographics, cardiovascular risk factors, lifestyle factors, and MSG_CGM_ (model 2 + MSG_CGM_, B: −0.071 10^−3^/kPa [−1.204, 1.063], *p* = 0.903). CV_CGM_ and TIR_CGM_ ≥ 70% were also not associated with carDC (ESM Table 4 and 6). Inconsistently, TIR_CGM_ was independently associated with carDC (model 2, B per 10% TIR_CGM_: −0.350 10^−3^/kPa [−0.646, −0.055], *p* = 0.020; ESM Table 5).

### Daily GV and arterial structure

**Fig. 2:**
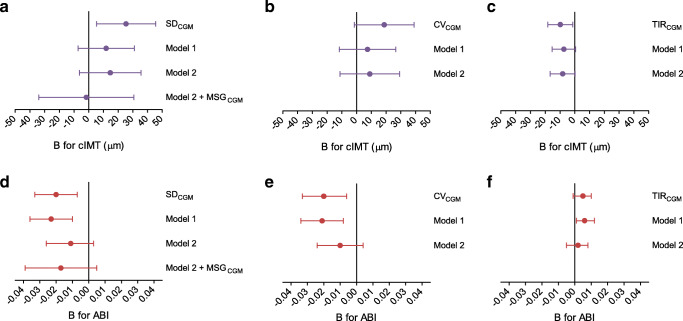
Multivariable-adjusted associations of SD_CGM_, CV_CGM_ and TIR_CGM_ with measures of arterial structure. Regression coefficients (B) indicate the mean difference (95% CI) associated with 1 mmol/l increase in SD_CGM_ or 10% increase in CV_CGM_ or TIR_CGM_. (**a**–**c**) Associations with cIMT and (**d**–**f**) associations with ABI. Model 1: adjusted for age, sex and education. Model 2: additionally adjusted for office systolic BP, mean heart rate (in case of ABI only), BMI, smoking status, alcohol use, total-to-HDL-cholesterol levels and use of antihypertensive and lipid-modifying drugs. Model 2 + MSG_CGM_: additionally adjusted for mean sensor glucose

Figure 2 and ESM Table 3 show the associations of SD_CGM_ with cIMT and ABI. SD_CGM_ and cIMT were not associated after adjustment for all potential confounders and MSG_CGM_ (model 2 + MSG_CGM_, B: −1.648 μm [−33.984, 30.688], *p* = 0.920). While CV_CGM_ and TIR_CGM_ were not independently associated with cIMT (ESM Table 4 and 5), TIR_CGM_ ≥ 70% was (model 2: B: −63.722 [−115.422, −12.023], *p* = 0.016; ESM Table 6).

Higher SD_CGM_ was statistically significantly associated with lower ABI after adjustment for demographics, but not after further adjustment for cardiovascular risk and lifestyle factors (model 2, B: −0.011 [−0.026, 0.003], *p* = 0.126). Adjustment for MSG_CGM_ numerically altered the regression coefficient but did not affect statistical significance (model 2 + MSG_CGM_, B: −0.017 [−0.039, 0.005], *p* = 0.121). Although CV_CGM_ and TIR_CGM_ were not independently associated with ABI (ESM Tables 4 and 5), TIR_CGM_ ≥ 70% was (model 2, B: 0.041 [0.004, 0.077], *p* = 0.030; ESM Table 6).

### Daily GV and circumferential wall stress

After full adjustment, SD_CGM_ was not associated with CWS_mean_ (model 2, B: 0.077 kPa [−1.313, 1.467], *p* = 0.913; ESM Table 3) or CWS_puls_ (model 2, B: −0.202 kPa [−1.019, 0.614], *p* = 0.627; ESM Table 3). Further adjustment for MSG_CGM_ did not materially alter the results. CV_CGM_ and TIR_CGM_ were not independently associated with CWS_mean_ and CWS_puls_ (ESM Tables 4 and 5).

### Interaction analyses

ESM Table 7 shows all *P*_interaction_ values for the associations between SD_CGM_ and the arterial outcome measures. A statistically significant *P*_interaction_ for age was only observed for the association between SD_CGM_ and cIMT (*p* = 0.044). The association between SD_CGM_ and cIMT was stronger in women (ESM Table 8). Age and type 2 diabetes status did not modify any of the studied associations (ESM Tables 7 and 9).

### Additional analyses

In general, the main results were not altered by replacement of MSG_CGM_ with GMS, HbA_1c_ or fasting plasma glucose (ESM Table 10); additional adjustment for physical activity and diet (ESM Table 11) or for eGFR, urinary albumin excretion, and history of CVD (ESM Table 12); replacement of office systolic BP with ambulatory systolic BP (ESM Table 13); or exclusion of individuals with type 1 diabetes (ESM Table 14). The associations of SD_CGM_ with arterial measures were, in general, slightly stronger after exclusion of individuals with CGM data gaps or with a suboptimal CGM recording period (ESM Tables 15 and 16). Exclusion of individuals who underwent CGM as part of a ‘catch-up visit’ substantially altered the associations between SD_CGM_ and the arterial measures (ESM Table 17). ESM Table 18 provides the associations of MSG_CGM_ with the arterial measures. Last, ESM Table 19 shows the effects of different degrees of ridge regression penalisation on the studied associations. In case of ABI, slight regularisation (λ = 0.11) reversed the st.β of MSG_CGM_.

## Discussion

In the present study, we investigated the cross-sectional associations of daily GV with several arterial outcome variables in a relatively large population of individuals who underwent more than 48 h of CGM. Our study has two main findings. First, greater GV was linearly associated with higher cf-PWV, the gold-standard measure to assess aortic stiffness, irrespective of demographics, cardiovascular risk factors and lifestyle factors. The observed association between SD_CGM_ and cf-PWV was corroborated by our CV_CGM_ and TIR_CGM_ results. Notably, SD_CGM_ and MSG_CGM_ contributed to a similar extent to the association with cf-PWV, which suggests an equivalent pathophysiological relevance to aortic stiffness. Second, we established no consistent independent associations between indices of daily GV and the other investigated arterial measures.

Our main analyses were performed in a study population that comprises the complete spectrum of daily GV (i.e., individuals with NGM, prediabetes, type 2 diabetes and type 1 diabetes). This approach is justified by the substantial overlap in CGM-derived indices between GMS groups, which can be appreciated from ESM Table 2, ESM Figs 2–4, and a recent publication on this cohort [31], and has several advantages over subgroup analyses, such as more statistical power [46] and less range restriction [47]. In addition, because no effect modification by type 2 diabetes status was observed (ESM Table 7), stratification was not indicated. Further, the linearity of the observed associations between daily GV and arterial measures is consistent with work on the ‘ticking clock hypothesis’, which postulates that hyperglycaemia-induced damage is a continuous process that starts in prediabetes, progresses with the onset of type 2 diabetes, and continues during type 2 diabetes [48, 49].

Few studies have investigated the association of CGM-measured GV with arterial measures [20–22] in concert with sufficient adjustment for potential confounders [19]. Lu et al. did not establish an association of GV with cIMT [19], which is in line with our cIMT results. Recently, we observed that the incremental glucose peak, an OGTT-based proxy of daily GV [31], was statistically significantly associated with higher cf-PWV and CWS_mean_, but not with carDC, cIMT and CWS_puls_ [25]. Notably, our current findings are corroborated by this larger study, as the directions of the regression coefficients generally correspond, and in both instances the strongest association was found with cf-PWV. We presume that discrepancies in statistical significance are largely attributable to the almost threefold sample size differences of our previous (*n* = 1849–1978) and current study populations (*n* = 643–816). Although Lu et al. previously reported on the relation between TIR_CGM_ and cIMT [19], we are the first to establish a statistically significant association of TIR_CGM_ with cf-PWV.

We present – as the primary analysis – MSG_CGM_-adjusted associations with SD_CGM_, and – as secondary analyses – associations with the intrinsically MSG_CGM_-adjusted index CV and with TIR_CGM_, which inversely reflects both mean blood glucose levels and GV [29]. Because they are strongly correlated, it is both necessary and complex to disentangle the effects of glucose fluctuations (i.e., SD_CGM_) and mean glucose (i.e., MSG_CGM_) [18]. The strong correlation between SD_CGM_ and MSG_CGM_ (rho = 0.69), the substantial increase (121–139%) in VIF from model 2 to model 2 + MSG_CGM_ (ESM Table 3), and the opposite directions of the regression coefficients of SD_CGM_ and MSG_CGM_ (e.g., ABI) all indicate multicollinearity [41]. Previous studies on other potential consequences of GV encountered similar contrariety [50, 51], but did not sufficiently address this point. We employed ridge regression to partially counter the potential adverse effects of multicollinearity, thereby allowing for better comparison of SD_CGM_ and MSG_CGM_ (Table 2). Notably in case of ABI, slight regularisation (λ = 0.11) reversed the st.β of MSG_CGM_ (ESM Table 19). Interestingly, the relative contributions of SD_CGM_ and MSG_CGM_ differed per measure. In the case of cf-PWV, the estimates were similar, which is corroborated by its independent association with CV_CGM_ and TIR_CGM_.

The biological mechanisms that mediate the relationship between GV and aortic stiffness require further elucidation. Several studies observed that greater GV augments inflammation and oxidative stress [52, 53]. This could promote the formation of advanced glycation end-products (AGEs) [54], which have been suggested to induce arterial stiffening by accumulating in the arterial wall and forming cross-links between elastin and collagen [3–5]. An association of tissue and circulating AGEs has, thus far, only been reported with cf-PWV [55, 56], which might explain our contrasting findings for the structurally different aorta (i.e., cf-PWV) and carotid artery (i.e., carDC, cIMT). In addition, cultured human fibroblasts synthesised more collagen during intermittently high glucose concentrations than during stable hyperglycaemia [57]. Higher GV could, thus, lead to higher aortic stiffness by altering the elastin:collagen ratio. Additionally, large-artery endothelial dysfunction may, in part, explain the association between daily GV and cf-PWV [5, 58]. Further, not only higher glucose peaks but also more pronounced glucose nadirs could contribute to CVD development [59]. Recurrent hypoglycaemia has, for example, been shown to negatively affect certain preclinical vascular measures in individuals with type 1 diabetes [60].

Aortic stiffness, assessed via cf-PWV, is an independent determinant of CVD, cardiovascular mortality and all-cause mortality [26–28]. We found that cf-PWV was 0.27–0.41 m/s higher per SD_CGM_ unit (mmol/l) increase in the final regression models (i.e., model 2, model 2 + MSG_CGM_), which corresponds with 3–4 years of vascular ageing [61]. Hence, the 0.8 mmol/l SD_CGM_ difference between the first and third SD_CGM_ tertile (Table 1) can be translated to a 2- or 3-year vascular ageing difference, which closely matches our recent findings on the OGTT-based incremental glucose peak [25]. Moreover, with every 10% higher TIR_CGM,_ cf-PWV was 0.15 m/s lower, which equals minus 18 months of vascular ageing [61]. After full adjustment, a TIR_CGM_ ≥ 70% corresponded to a 1.10 m/s lower cf-PWV, an 11-year vascular ageing difference [61]. This statistically significant association remained after further adjustment for HbA_1c_ (ESM Table 6), which strengthens the recommendations from the International Consensus on TIR_CGM_ [43]. Prospective studies should further explore the observed association with aortic stiffness. If confirmative, it would be justified to study whether interventions that specifically target CGM-measured GV or TIR_CGM_ (e.g., closed-loop insulin delivery systems) can improve CVD risk or incidence [16, 62].

This study has strengths and limitations. Strengths include: (1) the use of the gold-standard methods for daily GV quantification [17]; (2) the use of several, state-of-the-art arterial outcome measures; (3) the extensive participant characterisation, which enabled adjustment for a broad array of possible confounders; (4) the additional use of ridge regression, which allowed us to partly address multicollinearity between SD_CGM_ and MSG_CGM_; and (5) the robustness of the results, i.e., the overall consistency of several sensitivity analyses, in particular for cf-PWV.

Our study has specific limitations. First, a relatively large number of individuals were excluded because of missing outcome data (ESM Fig. 1). Although the study populations were generally comparable (ESM Table 1), the smaller sample size of the cf-PWV study population negatively impacted statistical power. Second, most of the individuals with diabetes had relatively well-controlled glycaemic indices [31]. The consequent range restriction in the upper SD_CGM_ and lower TIR_CGM_ spectrum may have biased the regression estimates towards null [47]. Third, the strength of the associations may have been additionally underestimated because of individuals who underwent CGM as a catch-up visit (*n* = 249; 29.2%) [63], as for these there was a median time of 2.1 years between CGM and the other measurements [31]. While the associations were also investigated in newly recruited individuals only (ESM Table 17), their applicability is substantially hampered by the smaller sample size and different GMS distribution (i.e., lower number of individuals with prediabetes and type 2 diabetes) of the study populations. Fourth, because of the cross-sectional design of our study, we are unable to rule out reverse causality. For example, as greater arterial stiffness has been associated with incident diabetes [64], it could increase GV. Fifth, it could be argued that adjustment for multiple testing would be required in our study [65]. However, we regarded the consequently higher chance of type 2 error undesirable [65, 66], especially in the context of a CGM-based study, which commonly has a relatively small sample size because of the costliness and relative invasiveness of CGM [18]. Further, it would be overly strict to enforce adjustment based on the determinants used, since SD_CGM_, CV_CGM_ and TIR_CGM_ are conceptually and statistically related [10, 29]. Sixth, our study population is predominately Caucasian, which might limit the generalisability of our results to other populations. Last, although the models were adjusted for many cardiovascular risk and lifestyle factors, residual confounding could still be present.

Our findings support the concept that greater daily GV and lower TIR_CGM_ are determinants of worse aortic stiffness, but do not support this for other arterial measures. Interestingly, the fully adjusted associations of SD_CGM_ and MSG_CGM_ with cf-PWV were comparable. Taken together, this study further underscores the pathophysiological relevance of daily GV, irrespective of mean glycaemia, in the context of macrovascular complications. Future studies should explore this association prospectively and assess whether interventions that specifically target CGM-measured GV or TIR_CGM_ can prevent CVD.

## Supplementary Information

ESM (PDF 1101 kb)

## Data Availability

Data are available from The Maastricht Study for any researcher who meets the criteria for access to confidential data; the corresponding author may be contacted to request data.

## Abbreviations

AGEs: Advanced glycation end-products
ABI: Ankle–brachial index
B: Regression coefficient
braPP: Brachial pulse pressure
carDC: Carotid distensibility coefficient
cf-PWV: Carotid–femoral pulse wave velocity
cIMT: Carotid intima–media thickness
CGM: Continuous glucose monitoring
CV_CGM_: CGM-assessed CV
CWS_mean_: Mean circumferential wall stress
CWS_puls_: Pulsatile circumferential wall stress
FPG: Fasting plasma glucose
GMS: Glucose metabolism status
GV: Glucose variability
IAD: Interadventitial diameter
LD: Carotid lumen diameter
MAP: Mean arterial pressure
MSG_CGM_: CGM-assessed mean sensor glucose
NGM: Normal glucose metabolism
SD_CGM_: CGM-assessed standard deviation
st.β: Standardised regression coefficient
TIR_CGM_: CGM-assessed time in range
VIF: Variance inflation factor
