# Supplementary material for: Greater daily glucose variability and lower time in range assessed with continuous glucose monitoring are associated with greater aortic stiffness: The Maastricht Study
Source: Diabetologia. 2021 May 15;64(8):1880–92. doi: 10.1007/s00125-021-05474-8 (PMC8245390; doi:10.1007/s00125-021-05474-8)
Supplement: Supplementary file 1 — (PDF 1101 kb) [file 125_2021_5474_MOESM1_ESM.pdf]

ESM to ‘Greater daily glucose variability and lower time in range are associated with greater aortic stiffness: The Maastricht Study’

ESM Methods

Ridge regression

The method used in R package glmnet (version 4.0.2) estimates regression coefficients according to the formula: $Ridge = RSS + \frac{1}{n} * \lambda * \sum_{j=1}^{p} \beta_j^2$, where RSS is the residual sum of squares, n is the sample size, λ is the chosen amount of penalization, and $\sum_{j=1}^{p} \beta_j^2$ represents the sum of all squared regression coefficients.

ESM Table 1. Participant characteristics of the different study populations and the number of missing values

Characteristic	Complete data on potential confounders (n=825)	Missing data	cf-PWV (n=643)	Missing data	carDC, CWS (n=725)	Missing data	cIMT (n=726)	Missing data	ABI (n=816)	Missing data
**Demographics**										
Age, y	59.7 ± 8.6	0	60.0 ± 8.5	0	60.1 ± 8.6	0	60.1 ± 8.6	0	59.7 ± 8.6	0
Women	403 (48.8)	0	295 (45.9)	0	350 (48.3)	0	351 (48.3)	0	398 (48.8)	0
Educational level (low/medium/high), n	261/231/333	0	197/184/262	0	226/211/288	0	226/211/289	0	256/227/333	0
Educational level (low/medium/high), %	31.6/28.0/40.4		30.6/28.6/40.7		31.2/29.1/39.7		31.1/29.1/39.8		31.4/27.8/40.8	
**Glycaemic parameters**										
Glucose metabolism status										
NGM/PreD/T2D/T1D, n	455/179/189/2	0	333/148/160/2	0	384/169/171/1	0	384/169/172/1	0	454/175/185/2	0
NGM/PreD/T2D/T1D, %	55.2/21.7/22.9/0.2		51.8/23.0/24.9/0.3		53.0/23.3/23.6/0.1		52.9/23.3/23.7/0.1		55.6/21.4/22.7/0.2	
Newly diagnosed T2D, n	68 (8.2)	0	57 (8.9)	0	59 (8.1)	0	59 (8.1)	0	68 (8.3)	0
Fasting plasma glucose, mmol/L	5.4 [5.0-6.2]	0	5.5 [5.0-6.4]	0	5.5 [5.0-6.3]	0	5.5 [5.0-6.3]	0	5.4 [5.0-6.2]	0
2-hour post-load glucose, mmol/L	6.6 [5.2-9.1]	39	6.9 [5.3-9.4]	33	6.8 [5.3-9.3]	39	6.9 [5.3-9.3]	39	6.6 [5.2-9.1]	39
MSG_{CGM}, mmol/L	6.1 [5.7-6.7]	0	6.1 [5.7-6.8]	0	6.1 [5.7-6.7]	0	6.1 [5.7-6.7]	0	6.1 [5.7-6.7]	0
SD_{CGM}, mmol/L	0.84 [0.68-1.19]	0	0.86 [0.69-1.22]	0	0.86 [0.69-1.21]	0	0.86 [0.69-1.21]	0	0.84 [0.68-1.17]	0
CV_{CGM}, %	14.0 [11.6-17.6]	0	14.3 [11.6-17.9]	0	14.3 [11.7-17.9]	0	14.3 [11.7-17.9]	0	14.0 [11.6-17.6]	0
TIR_{CGM}, %	99.8 [97.3-100.0]	0	99.7 [96.5-100.0]	0	99.7 [96.8-100.0]	0	99.7 [96.8-100.0]	0	99.8 [97.6-100.0]	0
HbA_{1c}, %	5.5 [5.3-5.9]	0	5.6 [5.4-5.9]	0	5.6 [5.3-5.9]	0	5.6 [5.4-5.9]	0	5.5 [5.3-5.9]	0
HbA_{1c}, mmol/mol	37.0 [34.0-41.0]	0	38.0 [35.0-41.0]	0	38.0 [35.0-41.0]	0	38.0 [35.0-41.0]	0	37.0 [34.0-41.0]	0
Diabetes medication use, n	106 (12.8)	0	90 (14.0)	0	98 (13.5)	0	99 (13.6)	0	102 (12.5)	0
Insulin	21 (2.5)		18 (2.8)		17 (2.3)		17 (2.3)		20 (2.5)	
Metformin	100 (12.1)		85 (13.2)		94 (13.0)		95 (13.1)		97 (11.9)	
Sulfonylureas	21 (2.5)		19 (3.0)		20 (2.8)		20 (2.8)		21 (2.6)	
GLP-1 analogs	4 (0.5)		4 (0.6)		3 (0.4)		3 (0.4)		4 (0.5)	
DDP-4 inhibitors	1 (0.1)		1 (0.2)		1 (0.1)		1 (0.1)		1 (0.1)	
SGLT-2 inhibitors	1 (0.1)		1 (0.2)		1 (0.1)		1 (0.1)		1 (0.1)	
**Lifestyle factors**										
BMI, kg/m^2	27.2 ± 4.4	0	27.3 ± 4.4	0	27.2 ± 4.4	0	27.2 ± 4.4	0	27.0 ± 4.2	0
Waist circumference (men), cm	102.4 ± 11.8	0	102.7 ± 11.8	0	102.5 ± 11.9	0	102.5 ± 11.9	0	102.1 ± 11.5	0
Waist circumference (women), cm	90.7 ± 12.2	0	91.0 ± 11.9	0	90.7 ± 12.1	0	90.7 ± 12.1	0	90.4 ± 12.0	0
Physical activity, hours/week	12.3 [7.5-18.3]	95	12.0 [7.5-18.3]	63	12.3 [7.5-18.4]	81	12.3 [7.5-18.5]	81	12.3 [7.5-18.4]	92
Dutch healthy diet index, (range: 0-150)	83.6 ± 16.1	200	83.4 ± 16.1	114	83.6 ± 15.8	154	83.6 ± 15.9	154	83.6 ± 16.0	199
Alcohol use (none/low/high), n	145/529/151	0	114/410/119	0	130/465/130	0	130/466/130	0	143/523/150	0
Alcohol use (none/low/high), %	17.6/64.1/18.3		17.7/63.8/18.5		17.9/64.1/19.9		17.9/64.2/19.9		17.5/64.1/18.4	
Smoking (never/former/current), n	321/402/102	0	254/310/79	0	278/356/91	0	279/356/91	0	317/397/102	0
Smoking (never/former/current), %	38.9/48.7/12.4		39.5/48.2/12.3		38.3/49.1/12.6		38.4/49.0/12.5		38.8/48.7/12.5	
**Cardiovascular risk factors**										
History of CVD	125 (15.2)	2	102 (15.9)	2	109 (15.1)	2	109 (15.1)	2	122 (15.0)	2
Office systolic BP, mmHg	133.2 ± 18.0	0	133.6 ± 18.1	0	133.5 ± 18.1	0	133.5 ± 18.1	0	133.1 ± 18.0	0

Office diastolic BP, mmHg	75.1 ± 10.2	0	75.3 ± 10.5	0	75.2 ± 10.2	0	75.2 ± 10.2	0	75.0 ± 10.2	0
Mean arterial pressure, mmHg	97.0 ± 10.8	0	97.0 ± 10.8	0	97.2 ± 10.9	0	97.2 ± 10.9	0	97.0 ± 10.8	0
Mean heart rate, beats/minute	61.0 ± 8.7	0	61.1 ± 8.7	0	61.1 ± 8.6	0	61.1 ± 8.6	0	60.9 ± 8.7	0
Antihypertensive medication use, n	290 (35.2)	0	235 (36.5)	0	261 (36.0)	0	262 (36.1)	0	284 (34.8)	0
Total-to-HDL cholesterol ratio	3.5 [2.8-4.3]	0	3.5 [2.9-4.4]	0	3.5 [2.8-4.3]	0	3.5 [2.8-4.3]	0	3.5 [2.8-4.3]	0
Triglycerides, mmol/L	1.3 [0.9-1.8]	0	1.3 [0.9-1.8]	0	1.3 [0.9-1.8]	0	1.3 [0.9-1.8]	0	1.3 [0.9-1.7]	0
Lipid-modifying medication use, n	205 (24.8)	0	171 (26.6)	0	187 (25.8)	0	187 (25.8)	0	201 (24.6)	0
eGFR, mL/min/1.73 m^2	80.5 ± 13.7	0	80.5 ± 13.8	0	80.1 ± 13.7	0	80.1 ± 13.7	0	80.5 ± 13.7	0
Albuminuria, n	63 (7.9)	3	52 (8.1)	3	59 (8.2)	3	59 (8.1)	3	63 (7.8)	3
**Outcome measures**										
cf-PWV, m/s	8.8 ± 2.2	182	8.8 ± 2.2	0	8.8 ± 2.2	125	8.8 ± 2.2	125	8.8 ± 2.2	179
carDC, 10^{-3}/kPa	15.8 ± 6.0	100	16.0 ± 6.1	43	15.8 ± 6.0	0	15.8 ± 6.0	1	15.8 ± 6.0	99
cIMT, µm	890.5 ± 152.8	100	891.9 ± 151.6	43	890.5 ± 152.8	0	890.5 ± 152.7	0	891.0 ± 153.3	99
ABI	1.14 ± 0.11	8	1.14 ± 0.12	8	1.14 ± 0.11	7	1.14 ± 0.11	7	1.13 ± 0.11	0
ABI < 0.9, n	24 (2.9)		16 (2.5)		20 (2.8)		20 (2.8)		24 (2.9)	
CWS_{mean}, kPa	44.0 [37.9-50.6]	100	43.7 [37.7-50.6]	43	44.0 [37.9-50.6]	0	44.0 [37.9-50.6]	1	44.0 [37.9-50.6]	99
CWS_{puls}, kPa	22.5 [19.1-27.0]	100	22.4 [19.0-26.8]	43	22.5 [19.1-27.0]	0	22.5 [19.1-27.0]	1	22.5 [19.0-27.0]	99

Data are reported as mean ± SD, median [interquartile range], or number (percentage %) as appropriate. Data represent the study population of participants with complete data on determinant and confounders or on determinant, outcome and confounders (in case of cf-PWV; carDC, CWS; cIMT; and ABI).

NGM, normal glucose metabolism; PreD, prediabetes; T2D, type 2 diabetes; T1D, type 1 diabetes, MSG_{CGM}, mean sensor glucose, SD_{CGM}, standard deviation; CV_{CGM}, coefficient of variation; TIR_{CGM}, time in range; HbA1c glycated hemoglobin A1c; GLP-1 glucagon-like peptide-1; DPP-4 dipeptidase-4; SGLT2, sodium/glucose cotransporter 2; BMI, body mass index; CVD, cardiovascular disease; BP, blood pressure; HDL high-density lipoprotein; eGFR, estimated glomerular filtration rate; CWS_{mean}, mean circumferential wall stress; CWS_{puls}, pulsatile circumferential wall stress.

ESM Table 2. GMS-stratified distributions of CGM-derived indices in the ankle-brachial index study population

**CGM-derived indices***		**NGM (n=454)**	**PreD (n=175)**	**T2D (n=185)**
MSG_{CGM}, mmol/L	Median [IQR]	5.8 [5.5 – 6.1]	6.2 [5.8 – 6.6]	7.5 [6.8 – 8.7]
	5th – 95th percentile	5.15 - 6.54	5.25 - 7.60	5.73 – 11.85
SD_{CGM}, mmol/L	Median [IQR]	0.72 [0.61 – 0.87]	0.89 [0.73 – 1.12]	1.55 [1.16 – 1.98]
	5th – 95th percentile	0.48 - 1.28	0.60 - 1.61	0.79 - 3.13
CV_{CGM}, %	Median [IQR]	12.6 [10.7 – 14.9]	14.7 [12.2 – 17.5]	19.3 [16.1 – 24.1]
	5th – 95th percentile	8.51 - 20.57	10.13 – 24.08	12.42 – 33.36
TIR_{CGM}, %	Median [IQR]	100.0 [99.5 – 100.0]	99.8 [98.5 – 100.0]	91.8 [79.1 – 98.3]
	5th – 95th percentile	95.91 – 100.0	87.96 – 100.0	24.18 – 100.0

GMS, glucose metabolism status; CGM, continuous glucose monitoring; NGM, normal glucose metabolism; PreD, prediabetes; T2D, type 2 diabetes; MSG_{CGM}, mean sensor glucose; SD_{CGM}, standard deviation; CV_{CGM}, coefficient of variation; TIR_{CGM}, time in range; IQR, interquartile range.

*Because of the small sample size (n=2), these values are not reported for individuals with type 1 diabetes.

ESM Table 3. Multivariable-adjusted associations of daily glucose variability (expressed as SD_{CGM}) with arterial outcome variables

Model	B (95%CI)	*P* value	VIF
Carotid femoral pulse wave velocity (cf-PWV), m/s (n=643)			
Crude	0.920 (0.636; 1.205)	< 0.001	1
Model 1	0.647 (0.384; 0.909)	< 0.001	1.044
Model 2*	0.413 (0.147; 0.679)	0.002	1.322
Model 2 + MSG_{CGM}	0.270 (-0.125; 0.666)	0.180	2.918
Carotid distensibility coefficient (carDC), 10^{-3}/kPa (n=725)			
Crude	-0.822 (-1.615; -0.029)	0.042	1
Model 1	-0.184 (-0.917; 0.549)	0.622	1.036
Model 2*	0.684 (-0.052; 1.420)	0.068	1.291
Model 2 + MSG_{CGM}	-0.071 (-1.204; 1.063)	0.903	3.070
Carotid intima-media thickness (cIMT), µm (n=726)			
Crude	25.441 (5.286; 45.595)	0.013	1
Model 1	11.907 (-7.253; 31.066)	0.223	1.036
Model 2†	14.679 (-6.257; 35.615)	0.169	1.286
Model 2 + MSG_{CGM}	-1.648 (-33.984; 30.688)	0.920	3.071
Ankle-brachial index (ABI) (n=816)			
Crude	-0.020 (-0.033; -0.007)	0.003	1
Model 1	-0.023 (-0.036; -0.010)	< 0.001	1.048
Model 2†	-0.011 (-0.026; 0.003)	0.126	1.339
Model 2 + MSG_{CGM}	-0.017 (-0.039; 0.005)	0.121	3.001
Mean circumferential wall stress (CWS_{mean}), kPa (n=725)			
Crude	1.530 (0.183; 2.877)	0.026	1
Model 1	1.009 (-0.320; 2.338)	0.136	1.036
Model 2‡	0.077 (-1.313; 1.467)	0.913	1.287
Model 2 + MSG_{CGM}	-1.126 (-3.271; 1.019)	0.303	3.070
Pulsatile circumferential wall stress (CWS_{puls}), kPa (n=725)			
Crude	1.551 (0.690; 2.413)	< 0.001	1
Model 1	1.014 (0.161; 1.867)	0.020	1.036
Model 2*	-0.202 (-1.019; 0.614)	0.627	1.291
Model 2 + MSG_{CGM}	-0.602 (-1.862; 0.658)	0.349	3.070

Regression coefficients (B) indicate the mean difference (95% confidence interval) associated with 1 unit (mmol/L) increase of SD_{CGM}. Crude: SD_{CGM}. Model 1: adjusted for age, sex, and education level. Model 2: additionally adjusted for mean arterial pressure (*), office systolic blood pressure (†) or brachial pulse pressure (‡), heart rate (in case of cf-PWV and ABI only), body mass index, smoking status, alcohol use, total-to-HDL cholesterol levels, and use of antihypertensive and lipid-modifying drugs. Model 2 + MSG_{CGM}: additionally adjusted for mean sensor glucose. VIF, variance inflation factor

ESM Table 4. Multivariable-adjusted associations of daily glucose variability (expressed as CV_{CGM}) with arterial outcome variables

Model	B (95%CI)	*P* value
	Carotid femoral pulse wave velocity (cf-PWV), m/s (n=643)	
Crude	0.732 (0.443; 1.021)	< 0.001
Model 1	0.488 (0.223; 0.752)	< 0.001
Model 2*	0.303 (0.046; 0.559)	0.021
	Carotid distensibility coefficient (carDC), 10^{-3}/kPa (n=725)	
Crude	-0.790 (-1.587; 0.006)	0.052
Model 1	-0.160 (-0.893; 0.573)	0.669
Model 2*	0.274 (-0.436; 0.984)	0.449
	Carotid intima-media thickness (cIMT), µm (n=726)	
Crude	18.784 (-1.497; 39.064)	0.069
Model 1	7.487 (-11.684; 26.659)	0.443
Model 2†	9.021 (-11.182; 29.223)	0.381
	Ankle-brachial index (ABI) (n=816)	
Crude	-0.020 (-0.033; -0.006)	0.003
Model 1	-0.021 (-0.034; -0.008)	0.002
Model 2†	-0.010 (-0.024; 0.004)	0.166
	Mean circumferential wall stress (CWS_{mean}), kPa (n=725)	
Crude	0.494 (-0.863; 1.851)	0.475
Model 1	0.131 (-1.200; 1.462)	0.847
Model 2‡	-0.440 (-1.779; 0.899)	0.519
	Pulsatile circumferential wall stress (CWS_{puls}), kPa (n=725)	
Crude	0.919 (0.049; 1.789)	0.038
Model 1	0.436 (-0.419; 1.292)	0.317
Model 2*	-0.388 (-1.174; 0.398)	0.333

Regression coefficients (B) indicate the mean difference (95% confidence interval) associated with 10% increase in CV_{CGM}. Crude: CV_{CGM}. Model 1: adjusted for age, sex, and education level. Model 2: additionally adjusted for mean arterial pressure (*), office systolic blood pressure (†) or brachial pulse pressure (‡), heart rate (in case of cf-PWV and ABI only), body mass index, smoking status, alcohol use, total-to-HDL cholesterol levels, and use of antihypertensive and lipid-modifying drugs.

ESM Table 5. Multivariable-adjusted associations of time in range (TIR_{CGM}) with arterial outcome variables

Model	B (95%CI)	*P* value
	Carotid femoral pulse wave velocity (cf-PWV), m/s (n=643)	
Crude	-0.303 (-0.424; -0.182)	< 0.001
Model 1	-0.247 (-0.356; -0.139)	< 0.001
Model 2*	-0.145 (-0.252; -0.038)	0.008
	Carotid distensibility coefficient (carDC), 10^{-3}/kPa (n=725)	
Crude	0.107 (-0.227; 0.441)	0.529
Model 1	0.028 (-0.277; 0.333)	0.857
Model 2*	-0.350 (-0.646; -0.055)	0.020
	Carotid intima-media thickness (cIMT), µm (n=726)	
Crude	-9.743 (-18.211; -1.276)	0.024
Model 1	-7.380 (-15.336; 0.576)	0.069
Model 2†	-8.144 (-16.563; 0.275)	0.058
	Ankle-brachial index (ABI) (n=816)	
Crude	0.005 (-0.001; 0.010)	0.118
Model 1	0.006 (0.001; 0.012)	0.026
Model 2†	0.002 (-0.005; 0.008)	0.620
	Mean circumferential wall stress (CWS_{mean}), kPa (n=725)	
Crude	-0.785 (-1.349; -0.220)	0.007
Model 1	-0.564 (-1.116; -0.013)	0.045
Model 2‡	-0.179 (-0.739; 0.380)	0.530
	Pulsatile circumferential wall stress (CWS_{puls}), kPa (n=725)	
Crude	-0.552 (-0.915; -0.190)	0.003
Model 1	-0.433 (-0.788; -0.079)	0.017
Model 2*	0.049 (-0.280; 0.378)	0.768

Regression coefficients (B) indicate the mean difference (95% confidence interval) associated with 10% increase in TIR_{CGM}. Crude: TIR_{CGM}. Model 1: adjusted for age, sex, and education level. Model 2: additionally adjusted for mean arterial pressure (*), office systolic blood pressure (†) or brachial pulse pressure (‡), heart rate (in case of cf-PWV and ABI only), body mass index, smoking status, alcohol use, total-to-HDL cholesterol levels, and use of antihypertensive and lipid-modifying drugs.

ESM Table 6. Multivariable-adjusted associations of time in range above 70% (TIR_{CGM}≥70%) with arterial outcome variables

Model	B (95%CI)	*P* value
Carotid femoral pulse wave velocity (cf-PWV), m/s (n=643)		
Crude	-1.943 (-2.707; -1.179)	< 0.001
Model 1	-1.732 (-2.416; -1.048)	< 0.001
Model 2*	-1.098 (-1.745; -0.451)	0.001
Model 2 + HbA1c	-0.775 (-1.504; -0.047)	0.037
Carotid distensibility coefficient (carDC), 10^{-3}/kPa (n=725)		
Crude	0.618 (-1.513; 2.749)	0.569
Model 1	0.455 (-1.482; 2.393)	0.645
Model 2*	-1.759 (-3.583; 0.066)	0.059
Model 2 + HbA1c	-1.828 (-3.886; 0.230)	0.082
Carotid intima-media thickness (cIMT), µm (n=726)		
Crude	-69.366 (-123.377; -15.354)	0.012
Model 1	-62.914 (-113.401; -12.426)	0.015
Model 2†	-63.722 (-115.422; -12.023)	0.016
Model 2 + HbA1c	-48.116 (-106.285; 10.054)	0.105
Ankle-brachial index (ABI) (n=816)		
Crude	0.055 (0.018; 0.091)	0.003
Model 1	0.063 (0.028; 0.099)	0.001
Model 2†	0.041 (0.004; 0.077)	0.030
Model 2 + HbA1c	0.045 (0.004; 0.086)	0.037
Mean circumferential wall stress (CWS_{mean}), kPa (n=725)		
Crude	-4.480 (-8.087; -0.872)	0.015
Model 1	-3.400 (-6.909; 0.108)	0.057
Model 2‡	-1.394 (-4.834; 2.047)	0.427
Model 2 + HbA1c	-2.242 (-6.119; 1.635)	0.257
Pulsatile circumferential wall stress (CWS_{puls}), kPa (n=725)		
Crude	-2.686 (-5.006; -0.365)	0.023
Model 1	-2.224 (-4.481; 0.203)	0.053
Model 2*	0.523 (-1.502; 2.549)	0.612
Model 2 + HbA1c	0.374 (-1.911; 2.659)	0.748

Regression coefficients (B) indicate the mean difference (95% confidence interval) associated with 10% increase in TIR_{CGM}. Crude: TIR_{CGM}≥70%. Model 1: adjusted for age, sex, and education level. Model 2: additionally adjusted for mean arterial pressure (*), office systolic blood pressure (†) or brachial pulse pressure (‡), heart rate (in case of cf-PWV and ABI only), body mass index, smoking status, alcohol use, total-to-HDL cholesterol levels, and use of antihypertensive and lipid-modifying drugs. Model 2 + HbA1c: additionally adjusted for HbA1c.

ESM Table 7. P values for interaction for sex, age, and type 2 diabetes status for the associations between SD_{CGM} and arterial outcome variables

Outcome	Sex	Age	Diabetes status[a]	Type 2 diabetes status[b]
cf-PWV*	0.41	0.10	0.57	0.41
carDC*	0.07	0.69	0.96	0.93
cIMT†	**0.044**	0.94	0.34	0.36
ABI†	0.68	0.80	0.86	0.15
CWS_{mean}‡	0.06	0.60	0.54	0.46
CWS_{puls}*	0.07	0.67	0.99	0.90

All models were adjusted for age, education level, mean arterial pressure (*), office systolic blood pressure (†) or brachial pulse pressure (‡), heart rate (in case of cf-PWV and ABI only), body mass index, smoking status, alcohol use, total-to-HDL cholesterol levels, and use of antihypertensive and lipid-modifying drugs.

a For the associations with 'SD_{CGM}*diabetes status' as interaction term, the associations were additionally adjusted for diabetes status. Bold denotes statistical significance; b For the associations with 'SD_{CGM}*type 2 diabetes status' as interaction term, the associations were additionally adjusted for type 2 diabetes status. Bold denotes statistical significance.

ESM Table 8. Sex-stratified associations of daily glucose variability (expressed as SD_{CGM}) with carotid intima-media thickness

Model	B (95%CI)	*P* value	B (95%CI)	*P* value
	cIMT, men (n=375)		*cIMT, women (n=351)*	
Crude	3.693 (-23.829; 31.215)	0.792	51.562 (21.871; 81.254)	0.001
Model 1	-7.205 (-33.606; 19.197)	0.592	39.873 (11.918; 67.829)	0.005
Model 2	1.003 (-27.653; 29.659)	0.945	33.853 (2.814; 64.891)	0.033
Model 2 + MSG_{CGM}	-7.146 (-51.779; 37.487)	0.753	1.448 (-46.749; 49.644)	0.953

Regression coefficients (B) indicate the mean difference (95% confidence interval) associated with 1 unit (mmol/L) increase of SD_{CGM}. Crude: SD_{CGM}. Model 1: adjusted for age and education level. Model 2: additionally adjusted for office systolic blood pressure, body mass index, smoking status, alcohol use, total-to-HDL cholesterol levels, and use of antihypertensive and lipid-modifying drugs. Model 2 + MSG_{CGM}: additionally adjusted for mean sensor glucose.

ESM Table 9. Diabetes status-stratified associations of daily glucose variability (expressed as SD_{CGM}) with arterial outcome measures

Model	B (95%CI)	*P* value	B (95%CI)	*P* value
cf-PWV, m/s	*No diabetes (n=481)*		*Diabetes (n=162)*	
Crude	1.031 (0.450; 1.613)	0.001	0.271 (-0.260; 0.802)	0.316
Model 1	0.483 (-0.057; 1.023)	0.079	0.345 (-0.148; 0.839)	0.169
Model 2*	0.505 (-0.008; 1.017)	0.054	0.276 (-0.171; 0.723)	0.224
Model 2 + MSG_{CGM}	0.249 (-0.373; 0.872)	0.432	0.367 (-0.247; 0.982)	0.239
carDC, 10^{-3}/kPa	*No diabetes (n=553)*		*Diabetes (n=172)*	
Crude	-1.096 (-2.794; 0.602)	0.205	0.947 (-0.375; 2.270)	0.159
Model 1	0.820 (-0.727; 2.368)	0.298	0.576 (-0.733; 1.884)	0.386
Model 2*	1.164 (-0.278; 2.605)	0.113	0.868 (-0.322; 2.058)	0.152
Model 2 + MSG_{CGM}	0.965 (-0.779; 2.708)	0.278	-0.575 (-2.254; 1.103)	0.499
cIMT, µm	*No diabetes (n=553)*		*Diabetes (n=173)*	
Crude	69.320 (26.604; 112.037)	0.002	4.316 (-30.835; 39.467)	0.809
Model 1	36.178 (-4.282; 76.638)	0.080	13.354 (-19.747; 46.455)	0.427
Model 2†	37.967 (-3.157; 79.091)	0.070	13.855 (-19.914; 47.625)	0.419
Model 2 + MSG_{CGM}	18.148 (-31.489; 67.784)	0.473	-7.393 (-56.083; 41.297)	0.765
ABI	*No diabetes (n=629)*		*Diabetes (n=187)*	
Crude	-0.026 (-0.053; 0.001)	0.060	-0.014 (-0.040; 0.011)	0.289
Model 1	-0.023 (-0.049; 0.004)	0.091	-0.015 (-0.041; 0.011)	0.252
Model 2†	-0.013 (-0.039; 0.014)	0.354	-0.007 (-0.035; 0.020)	0.588
Model 2 + MSG_{CGM}	-0.013 (-0.045; 0.019)	0.425	-0.017 (-0.055; 0.020)	0.363
CWS_{mean}, kPa	*No diabetes (n=553)*		*Diabetes (n=172)*	
Crude	-1.280 (-3.991; 1.431)	0.354	-0.032 (-2.661; 2.124)	0.981
Model 1	-1.523 (-4.232; 1.187)	0.270	-0.434 (-2.992; 2.124)	0.738
Model 2‡	-1.508 (-4.094; 1.078)	0.253	-0.196 (-2.741; 2.349)	0.879
Model 2 + MSG_{CGM}	-0.955 (-4.083; 2.172)	0.549	-1.763 (-5.421; 1.895)	0.343
CWS_{puls}, kPa	*No diabetes (n=553)*		*Diabetes (n=172)*	
Crude	0.549 (-1.203; 2.300)	0.539	-0.344 (-1.970; 1.282)	0.677
Model 1	-0.558 (-2.295; 1.179)	0.528	-0.315 (-1.961; 1.330)	0.706
Model 2*	-0.876 (-2.412; 0.660)	0.263	-0.725 (-2.218; 0.768)	0.339
Model 2 + MSG_{CGM}	-0.698 (-2.556; 1.160)	0.461	-1.069 (-3.211; 1.072)	0.326

Regression coefficients (B) indicate the mean difference (95% confidence interval) associated with 1 unit (mmol/L) increase of SD_{CGM}. Crude: SD_{CGM}. Model 1: adjusted for age, sex, and education level. Model 2: additionally adjusted for mean arterial pressure (*), office systolic blood pressure (†) or brachial pulse pressure (‡), heart rate (in case of cf-PWV and ABI only), body mass index, smoking status, alcohol use, total-to-HDL cholesterol levels, and use of antihypertensive and lipid-modifying drugs. Model 2 + MSG_{CGM}: additionally adjusted for mean sensor glucose.

ESM Table 10. Multivariable-adjusted associations of daily glucose variability (expressed as SD_{CGM}) with arterial outcome variables after adjustment for glucose metabolism status, HbA_{1c}, or fasting plasma glucose instead of mean sensor glucose

Model	B (95%CI)	*P* value
Carotid femoral pulse wave velocity (cf-PWV), m/s (n=643)		
Model 2 + GMS	0.260 (-0.058; 0.579)	0.109
Model 2 + HbA_{1c}	0.244 (-0.081; 0.569)	0.140
Model 2 + FPG	0.294 (-0.014; 0.603)	0.061
Carotid distensibility coefficient (carDC), 10^{-3}/kPa (n=725)		
Model 2 + GMS	0.827 (-0.062; 1.716)	0.068
Model 2 + HbA_{1c}	0.792 (-0.116; 1.700)	0.087
Model 2 + FPG	0.687 (-0.157; 1.531)	0.111
Carotid intima-media thickness (cIMT), µm (n=726)		
Model 2 + GMS	22.231 (-3.061; 47.524)	0.085
Model 2 + HbA_{1c}	2.242 (-23.487; 27.972)	0.864
Model 2 + FPG	9.738 (-14.259; 33.735)	0.426
Ankle-brachial index (ABI) (n=816)		
Model 2 + GMS	-0.009 (-0.026; 0.008)	0.307
Model 2 + HbA_{1c}	-0.013 (-0.031; 0.004)	0.141
Model 2 + FPG	-0.009 (-0.025; 0.008)	0.290
Mean circumferential wall stress (CWS_{mean}), kPa (n=725)		
Model 2 + GMS	-0.670 (-2.349; 1.010)	0.434
Model 2 + HbA_{1c}	0.408 (-1.307; 2.123)	0.641
Model 2 + FPG	0.275 (-1.321; 1.871)	0.735
Pulsatile circumferential wall stress (CWS_{puls}), kPa (n=725)		
Model 2 + GMS	-0.868 (-1.853; 0.117)	0.084
Model 2 + HbA_{1c}	-0.130 (-1.138; 0.879)	0.801
Model 2 + FPG	-0.147 (-1.084; 0.791)	0.759

Regression coefficients (B) indicate the mean difference (95% confidence interval) associated with 1 unit (mmol/L) increase of SD_{CGM}. Model 2 + represents the fully adjusted model that was additionally adjusted for glucose metabolism status (GMS), HbA_{1c}, or fasting plasma glucose (FPG)

ESM Table 11. Multivariable-adjusted associations of SD_{CGM} with arterial outcome variables, additionally adjusted for physical activity and Dutch healthy diet adherence

Model	B (95%CI)	*P* value
Carotid femoral pulse wave velocity (cf-PWV), m/s (n=487)		
Crude	0.970 (0.639; 1.301)	< 0.001
Model 1	0.669 (0.356; 0.981)	< 0.001
Model 2*	0.487 (0.169; 0.804)	0.003
Model 3	0.487 (0.169; 0.805)	0.003
Model 3 + MSG_{CGM}	0.461 (-0.014; 0.935)	0.057
Carotid distensibility coefficient (carDC), 10^{-3}/kPa (n=519)		
Crude	-0.895 (-1.806; 0.017)	0.054
Model 1	-0.256 (-1.112; 0.601)	0.558
Model 2*	0.628 (-0.219; 1.475)	0.146
Model 3	0.641 (-0.209; 1.490)	0.139
Model 3 + MSG_{CGM}	0.866 (-0.461; 2.194)	0.200
Carotid intima-media thickness (cIMT), µm (n=520)		
Crude	18.139 (-5.039; 41.317)	0.125
Model 1	3.957 (-18.619; 26.534)	0.731
Model 2†	6.775 (-18.133; 31.683)	0.593
Model 3	7.294 (-17.684; 32.271)	0.566
Model 3 + MSG_{CGM}	-10.304 (-49.368; 28.760)	0.605
Ankle-brachial index (ABI) (n=562)		
Crude	-0.025 (-0.040; -0.009)	0.002
Model 1	-0.029 (-0.044; -0.013)	< 0.001
Model 2†	-0.014 (-0.031; 0.003)	0.115
Model 3	-0.014 (-0.031; 0.003)	0.112
Model 3 + MSG_{CGM}	-0.021 (-0.047; 0.005)	0.114
Mean circumferential wall stress (CWS_{mean}), kPa (n=519)		
Crude	2.785 (1.184; 4.386)	0.001
Model 1	1.952 (0.348; 3.556)	0.017
Model 2‡	1.242 (-0.445; 2.928)	0.149
Model 3	1.227 (-0.465; 2.918)	0.155
Model 3 + MSG_{CGM}	-0.040 (-2.688; 2.607)	0.976
Pulsatile circumferential wall stress (CWS_{puls}), kPa (n=519)		
Crude	2.024 (0.978; 3.071)	< 0.001
Model 1	1.414 (0.367; 2.462)	0.008
Model 2*	0.110 (-0.888; 1.109)	0.828
Model 3	0.059 (-0.938; 1.056)	0.908
Model 3 + MSG_{CGM}	-0.605 (-2.161; 0.951)	0.445

Regression coefficients (B) indicate the mean difference (95% confidence interval) associated 1 unit (mmol/L) increase of SD_{CGM}. Crude: SD_{CGM}. Model 1: adjusted for age, sex, and education level. Model 2: additionally adjusted for mean arterial pressure (*), office systolic blood pressure (†) or brachial pulse pressure (‡), heart rate (in case of cf-PWV and ABI only), body mass index, smoking status, alcohol use, total-to-HDL cholesterol levels, and use of antihypertensive and lipid-modifying drugs. Model 3: additionally adjusted for physical activity and Dutch healthy diet adherence. Model 3 + MSG_{CGM}: additionally adjusted for mean sensor glucose.

Of note, the alcohol component of the Dutch healthy diet adherence index was subtracted from the sum score prior to performing the regression analyses to avoid multicollinearity.

ESM Table 12. Multivariable-adjusted associations of SD_{CGM} with arterial outcome variables, additionally adjusted for history of cardiovascular disease, estimated glomerular filtration rate, and urinary albumin excretion

Model	B (95%CI)	*P* value
Carotid femoral pulse wave velocity (cf-PWV), m/s (n=638)		
Crude	0.885 (0.599; 1.171)	< 0.001
Model 1	0.609 (0.347; 0.871)	< 0.001
Model 2*	0.390 (0.124; 0.656)	0.004
Model 3	0.315 (0.048; 0.583)	0.021
Model 3 + MSG_{CGM}	0.225 (-0.169; 0.618)	0.263
Carotid distensibility coefficient (carDC), 10^{-3}/kPa (n=720)		
Crude	-0.832 (-1.631; -0.033)	0.041
Model 1	-0.187 (-0.925; 0.551)	0.620
Model 2*	0.668 (-0.072; 1.409)	0.077
Model 3	0.697 (-0.061; 1.455)	0.071
Model 3 + MSG_{CGM}	-0.015 (-1.167; 1.138)	0.980
Carotid intima-media thickness (cIMT), µm (n=721)		
Crude	25.173 (4.872; 45.474)	0.015
Model 1	11.635 (-7.671; 30.942)	0.237
Model 2†	14.680 (-6.391; 35.751)	0.172
Model 3	15.638 (-5.910; 37.187)	0.155
Model 3 + MSG_{CGM}	1.648 (-31.181; 34.477)	0.922
Ankle-brachial index (ABI) (n=811)		
Crude	-0.021 (-0.034; -0.008)	0.002
Model 1	-0.024 (-0.037; -0.011)	< 0.001
Model 2†	-0.012 (-0.027; 0.002)	0.094
Model 3	-0.007 (-0.022; 0.007)	0.333
Model 3 + MSG_{CGM}	-0.012 (-0.034; 0.009)	0.258
Mean circumferential wall stress (CWS_{mean}), kPa (n=720)		
Crude	1.430 (0.079; 2.782)	0.038
Model 1	0.911 (-0.423; 2.245)	0.180
Model 2‡	-0.036 (-1.431; 1.359)	0.960
Model 3	-0.129 (-1.556; 1.297)	0.859
Model 3 + MSG_{CGM}	-1.300 (-3.472; 0.872)	0.240
Pulsatile circumferential wall stress (CWS_{puls}), kPa (n=720)		
Crude	1.528 (0.661; 2.395)	0.001
Model 1	0.989 (0.131; 1.846)	0.024
Model 2*	-0.215 (-1.036; 0.605)	0.607
Model 3	-0.306 (-1.143; 0.532)	0.474
Model 3 + MSG_{CGM}	-0.787 (-2.062; 0.488)	0.226

Regression coefficients (B) indicate the mean difference (95% confidence interval) associated 1 unit (mmol/L) increase of SD_{CGM}. Crude: SD_{CGM}. Model 1: adjusted for age, sex, and education level. Model 2: additionally adjusted for mean arterial pressure (*), office systolic blood pressure (†) or brachial pulse pressure (‡), heart rate (in case of cf-PWV and ABI only), body mass index, smoking status, alcohol use, total-to-HDL cholesterol levels, and use of antihypertensive and lipid-modifying drugs. Model 3: additionally adjusted for history of cardiovascular disease, estimated glomerular filtration rate, and urinary albumin excretion. Model 3 + MSG_{CGM}: additionally adjusted for mean sensor glucose.

ESM Table 13. Multivariable-adjusted associations of SD_{CGM} with arterial structure adjusted for ambulatory systolic blood pressure instead of office systolic blood pressure

Model	B (95%CI)	*P* value
Carotid intima-media thickness (cIMT), µm (n=649)		
Crude	29.322 (7.349; 51.294)	0.009
Model 1	15.560 (-5.410; 36.530)	0.146
Model 2	18.314 (-4.281; 40.909)	0.112
Model 2 + MSG_{CGM}	8.592 (-25.341; 42.525)	0.619
Ankle-brachial index (ABI) (n=728)		
Crude	-0.026 (-0.040; -0.012)	< 0.001
Model 1	-0.030 (-0.044; -0.016)	< 0.001
Model 2	-0.019 (-0.034; -0.004)	0.015
Model 2 + MSG_{CGM}	-0.021 (-0.043; 0.001)	0.062

Regression coefficients (B) indicate the mean difference (95% confidence interval) associated 1 unit (mmol/L) increase of SD_{CGM}. Crude: SD_{CGM}. Model 1: adjusted for age, sex, and education level. Model 2: additionally adjusted for ambulatory systolic blood pressure and heart rate (in case of cf-PWV and ABI only), body mass index, smoking status, alcohol use, total-to-HDL cholesterol levels, and use of antihypertensive and lipid-modifying drugs. Model 2 + MSG_{CGM}: additionally adjusted for mean sensor glucose.

ESM Table 14. Multivariable-adjusted associations of SD_{CGM} with arterial outcome variables after exclusion of individuals with type 1 diabetes

Model	B (95%CI)	*P* value
Carotid femoral pulse wave velocity (cf-PWV), m/s (n=641)		
Crude	0.910 (0.619; 1.200)	< 0.001
Model 1	0.629 (0.362; 0.896)	< 0.001
Model 2*	0.402 (0.132; 0.673)	0.004
Model 2 + MSG_{CGM}	0.256 (-0.146; 0.658)	0.211
Carotid distensibility coefficient (carDC), 10^{-3}/kPa (n=724)		
Crude	-0.831 (-1.627; -0.034)	0.041
Model 1	-0.187 (-0.923; 0.549)	0.618
Model 2*	0.679 (-0.058; 1.416)	0.071
Model 2 + MSG_{CGM}	-0.068 (-1.203; 1.067)	0.906
Carotid intima-media thickness (cIMT), µm (n=725)		
Crude	25.389 (5.144; 45.634)	0.014
Model 1	11.724 (-7.511; 30.960)	0.232
Model 2†	14.708 (-6.267; 35.682)	0.169
Model 2 + MSG_{CGM}	-1.779 (-34.168; 30.610)	0.914
Ankle-brachial index (ABI) (n=814)		
Crude	-0.017 (-0.030; -0.003)	0.014
Model 1	-0.020 (-0.033; -0.007)	0.003
Model 2†	-0.007 (-0.022; 0.007)	0.313
Model 2 + MSG_{CGM}	-0.011 (-0.033; 0.011)	0.328
Mean circumferential wall stress (CWS_{mean}), kPa (n=724)		
Crude	1.527 (0.174; 2.880)	0.027
Model 1	1.026 (-0.308; 2.360)	0.132
Model 2‡	0.103 (-1.289; 1.495)	0.884
Model 2 + MSG_{CGM}	-1.170 (-3.317; 0.977)	0.285
Pulsatile circumferential wall stress (CWS_{puls}), kPa (n=724)		
Crude	1.532 (0.667; 2.397)	0.001
Model 1	0.999 (0.143; 1.855)	0.022
Model 2*	-0.209 (-1.027; 0.610)	0.617
Model 2 + MSG_{CGM}	-0.596 (-1.858; 0.666)	0.354

Regression coefficients (B) indicate the mean difference (95% confidence interval) associated with 1 unit (mmol/L) increase of SD_{CGM}. Crude: SD_{CGM}. Model 1: adjusted for age, sex, and education level. Model 2: additionally adjusted for mean arterial pressure (*), office systolic blood pressure (†) or brachial pulse pressure (‡), heart rate (in case of cf-PWV and ABI only), body mass index, smoking status, alcohol use, total-to-HDL cholesterol levels, and use of antihypertensive and lipid-modifying drugs. Model 2 + MSG_{CGM}: additionally adjusted for mean sensor glucose.

ESM Table 15. Multivariable-adjusted associations of daily glucose variability (expressed as SD_{CGM}) with arterial outcome variables after exclusion of individuals with CGM data gaps

Model	B (95%CI)	*P* value
Carotid femoral pulse wave velocity (cf-PWV), m/s (n=591)		
Crude	0.984 (0.671; 1.297)	< 0.001
Model 1	0.734 (0.446; 1.022)	< 0.001
Model 2*	0.501 (0.207; 0.796)	0.001
Model 2 + MSG_{CGM}	0.368 (-0.095; 0.831)	0.119
Carotid distensibility coefficient (carDC), 10^{-3}/kPa (n=672)		
Crude	-0.628 (-1.470; 0.214)	0.144
Model 1	-0.043 (-0.821; 0.736)	0.914
Model 2*	0.909 (0.127; 1.691)	0.023
Model 2 + MSG_{CGM}	0.139 (-1.102; 1.380)	0.826
Carotid intima-media thickness (cIMT), µm (n=673)		
Crude	20.012 (-1.404; 41.428)	0.067
Model 1	8.587 (-11.835; 29.009)	0.409
Model 2†	12.413 (-9.973; 34.798)	0.277
Model 2 + MSG_{CGM}	-2.126 (-37.762; 33.510)	0.907
Ankle-brachial index (ABI) (n=749)		
Crude	-0.023 (-0.037; -0.008)	0.002
Model 1	-0.026 (-0.040; -0.011)	< 0.001
Model 2†	-0.015 (-0.031; 0.001)	0.071
Model 2 + MSG_{CGM}	-0.026 (-0.051; 0.000)	0.047
Mean circumferential wall stress (CWS_{mean}), kPa (n=672)		
Crude	1.751 (0.311; 3.192)	0.017
Model 1	1.254 (-0.165; 2.672)	0.131
Model 2‡	0.180 (-1.309; 1.670)	0.812
Model 2 + MSG_{CGM}	-1.027 (-3.396; 1.341)	0.395
Pulsatile circumferential wall stress (CWS_{puls}), kPa (n=672)		
Crude	1.693 (0.773; 2.614)	< 0.001
Model 1	1.230 (0.315; 2.145)	0.008
Model 2*	-0.011 (-0.886; 0.864)	0.980
Model 2 + MSG_{CGM}	-0.277 (-1.668; 1.114)	0.696

Regression coefficients (B) indicate the mean difference (95% confidence interval) associated with 1 unit (mmol/L) increase of SD_{CGM}. Crude: SD_{CGM}. Model 1: adjusted for age, sex, and education level. Model 2: additionally adjusted for mean arterial pressure (*), office systolic blood pressure (†) or brachial pulse pressure (‡), heart rate (in case of cf-PWV and ABI only), body mass index, smoking status, alcohol use, total-to-HDL cholesterol levels, and use of antihypertensive and lipid-modifying drugs. Model 2 + MSG_{CGM}: additionally adjusted for mean sensor glucose.

ESM Table 16. Multivariable-adjusted associations of daily glucose variability (expressed as SD_{CGM}) with arterial outcome variables after exclusion of individuals with a suboptimal CGM recording period

Model	B (95%CI)	*P* value
Carotid femoral pulse wave velocity (cf-PWV), m/s (n=620)		
Crude	0.931 (0.642; 1.221)	< 0.001
Model 1	0.649 (0.381; 0.917)	< 0.001
Model 2*	0.425 (0.151; 0.698)	0.002
Model 2 + MSG_{CGM}	0.299 (-0.109; 0.706)	0.151
Carotid distensibility coefficient (carDC), 10^{-3}/kPa (n=698)		
Crude	-0.711 (-1.519; 0.098)	0.085
Model 1	-0.017 (-0.765; 0.730)	0.964
Model 2*	0.782 (0.028; 1.537)	0.044
Model 2 + MSG_{CGM}	0.066 (-1.116; 1.248)	0.913
Carotid intima-media thickness (cIMT), µm (n=699)		
Crude	26.580 (6.011; 47.149)	0.011
Model 1	12.627 (-6.950; 32.204)	0.206
Model 2†	16.739 (-4.712; 38.190)	0.126
Model 2 + MSG_{CGM}	4.126 (-29.577; 37.829)	0.810
Ankle-brachial index (ABI) (n=785)		
Crude	-0.020 (-0.034; -0.007)	0.003
Model 1	-0.024 (-0.037; -0.010)	0.001
Model 2†	-0.011 (-0.026; 0.004)	0.163
Model 2 + MSG_{CGM}	-0.019 (-0.041; 0.004)	0.109
Mean circumferential wall stress (CWS_{mean}), kPa (n=698)		
Crude	1.514 (0.155; 2.872)	0.029
Model 1	0.944 (-0.396; 2.285)	0.167
Model 2‡	0.097 (-1.316; 1.510)	0.893
Model 2 + MSG_{CGM}	-1.544 (-3.759; 0.671)	0.172
Pulsatile circumferential wall stress (CWS_{puls}), kPa (n=698)		
Crude	1.579 (0.707; 2.452)	< 0.001
Model 1	0.996 (0.133; 1.859)	0.024
Model 2*	-0.175 (-1.006; 0.656)	0.679
Model 2 + MSG_{CGM}	-0.619 (-1.923; 0.685)	0.352

Regression coefficients (B) indicate the mean difference (95% confidence interval) associated with 1 unit (mmol/L) increase of SD_{CGM}. Crude: SD_{CGM}. Model 1: adjusted for age, sex, and education level. Model 2: additionally adjusted for mean arterial pressure (*), office systolic blood pressure (†) or brachial pulse pressure (‡), heart rate (in case of cf-PWV and ABI only), body mass index, smoking status, alcohol use, total-to-HDL cholesterol levels, and use of antihypertensive and lipid-modifying drugs. Model 2 + MSG_{CGM}: additionally adjusted for mean sensor glucose.

ESM Table 17. Multivariable-adjusted associations of daily glucose variability (expressed as SD_{CGM}) with arterial outcome variables after exclusion of individuals who underwent CGM as part of a 'catch-up visit'

Model	B (95%CI)	*P* value
Carotid femoral pulse wave velocity (cf-PWV), m/s (n=418)		
Crude	0.724 (0.337; 1.107)	< 0.001
Model 1	0.408 (0.062; 0.754)	0.021
Model 2*	0.188 (-0.160; 0.536)	0.289
Model 2 + MSG_{CGM}	-0.200 (-0.673; 0.273)	0.406
Carotid distensibility coefficient (carDC), 10^{-3}/kPa (n=500)		
Crude	-0.728 (-1.882; 0.425)	0.215
Model 1	0.147 (-0.904; 1.198)	0.784
Model 2*	0.521 (-0.513; 1.555)	0.323
Model 2 + MSG_{CGM}	-0.355 (-1.758; 1.047)	0.619
Carotid intima-media thickness (cIMT), µm (n=500)		
Crude	42.209 (12.158; 72.261)	0.006
Model 1	22.183 (-5.946; 50.312)	0.122
Model 2†	27.960 (-2.331; 58.251)	0.070
Model 2 + MSG_{CGM}	4.808 (-36.285; 45.902)	0.818
Ankle-brachial index (ABI) (n=577)		
Crude	0.000 (-0.019; 0.019)	0.998
Model 1	-0.004 (-0.022; 0.015)	0.684
Model 2†	0.011 (-0.009; 0.031)	0.266
Model 2 + MSG_{CGM}	-0.002 (-0.028; 0.025)	0.902
Mean circumferential wall stress (CWS_{mean}), kPa (n=500)		
Crude	-0.767 (-2.710; 1.176)	0.438
Model 1	-0.987 (-2.909; 0.936)	0.314
Model 2‡	-1.740 (-3.697; 0.217)	0.081
Model 2 + MSG_{CGM}	-1.867 (-4.531; 0.797)	0.169
Pulsatile circumferential wall stress (CWS_{puls}), kPa (n=500)		
Crude	0.434 (-0.824; 1.692)	0.498
Model 1	-0.139 (-1.382; 1.105)	0.827
Model 2*	-0.872 (-2.060; 0.315)	0.150
Model 2 + MSG_{CGM}	-0.473 (-2.088; 1.142)	0.565

Regression coefficients (B) indicate the mean difference (95% confidence interval) associated with 1 unit (mmol/L) increase of SD_{CGM}. Crude: SD_{CGM}. Model 1: adjusted for age, sex, and education level. Model 2: additionally adjusted for mean arterial pressure (*), office systolic blood pressure (†) or brachial pulse pressure (‡), heart rate (in case of cf-PWV and ABI only), body mass index, smoking status, alcohol use, total-to-HDL cholesterol levels, and use of antihypertensive and lipid-modifying drugs. Model 2 + MSG_{CGM}: additionally adjusted for mean sensor glucose.

ESM Table 18. Multivariable-adjusted associations of mean sensor glucose with arterial outcome variables

Model	B (95%CI)	*P* value
Carotid femoral pulse wave velocity (cf-PWV), m/s (n=643)		
Crude	0.437 (0.315; 0.560)	< 0.001
Model 1	0.298 (0.184; 0.412)	< 0.001
Model 2*	0.177 (0.057; 0.297)	0.004
Model 2 + SD_{CGM}	0.087 (-0.091; 0.265)	0.338
Carotid distensibility coefficient (carDC), 10^{-3}/kPa (n=725)		
Crude	-0.423 (-0.759; -0.087)	0.014
Model 1	-0.143 (-0.456; 0.171)	0.372
Model 2*	0.412 (0.089; 0.734)	0.012
Model 2 + SD_{CGM}	0.435 (-0.062; 0.933)	0.086
Carotid intima-media thickness (cIMT), µm (n=726)		
Crude	14.612 (6.085; 23.139)	0.001
Model 1	7.526 (-0.670; 15.722)	0.072
Model 2†	8.847 (-0.330; 18.024)	0.059
Model 2 + SD_{CGM}	9.398 (-4.793; 23.589)	0.194
Ankle-brachial index (ABI) (n=816)		
Crude	-0.005 (-0.011; 0.000)	0.068
Model 1	-0.008 (-0.014; -0.002)	0.006
Model 2†	-0.002 (-0.009; 0.004)	0.507
Model 2 + SD_{CGM}	0.004 (-0.0006 0.013)	0.477
Mean circumferential wall stress (CWS_{mean}), kPa (n=725)		
Crude	1.096 (0.528; 1.664)	< 0.001
Model 1	0.823 (0.256; 1.390)	0.005
Model 2‡	0.317 (-0.293; 0.927)	0.308
Model 2 + SD_{CGM}	0.694 (-0.248; 1.635)	0.149
Pulsatile circumferential wall stress (CWS_{puls}), kPa (n=725)		
Crude	0.905 (0.542; 1.268)	< 0.001
Model 1	0.657 (0.293; 1.020)	< 0.001
Model 2*	0.029 (-0.330; 0.388)	0.873
Model 2 + SD_{CGM}	0.230 (-0.323; 0.784)	0.414

Regression coefficients (B) indicate the mean difference (95% confidence interval) associated with 1 unit (mmol/L) increase of MSG_{CGM}. Crude: SD_{CGM}. Model 1: adjusted for age, sex, and education level. Model 2: additionally adjusted for mean arterial pressure (*), office systolic blood pressure (†) or brachial pulse pressure (‡), heart rate (in case of cf-PWV and ABI only), body mass index, smoking status, alcohol use, total-to-HDL cholesterol levels, and use of antihypertensive and lipid-modifying drugs. Model 2 + SD_{CGM}: additionally adjusted for standard deviation.

ESM Table 19. Regression coefficients of standard deviation and mean sensor glucose in the fully adjusted models with arterial outcome variables estimated with different degrees of penalization

VIF	SD (B, 95%CI)	SD_{CGM} (st.β, 95%CI)	*P* value	MSG_{CGM} (st.β, 95%CI)	*P* value
Carotid femoral pulse wave velocity (cf-PWV) (n=643)*					
Model 2 + MSG_{CGM} (λ=0)	0.259 (-0.127; 0.771)	0.070 (-0.037; 0.190)	0.198	0.055 (-0.086; 0.174)	0.426
Halfway (λ=0.04)	0.246 (-0.113; 0.723)	0.065 (-0.031; 0.183)	0.192	0.054 (-0.065; 0.174)	0.378
Model 2 (λ=0.11)	0.250 (-0.067; 0.663)	0.065 (-0.018; 0.167)	0.160	0.059 (-0.043; 0.164)	0.272
Carotid distensibility coefficient (carDC) (n=725)*					
Model 2 + MSG_{CGM} (λ=0)	-0.087 (-1.154; 1.088)	-0.008 (-0.106; 0.102)	0.864	0.095 (-0.024; 0.205)	0.110
Halfway (λ=0.04)	-0.069 (-1.179; 1.055)	-0.006 (-0.106; 0.095)	0.904	0.089 (-0.017; 0.201)	0.086
Model 2 (λ=0.12)	-0.035 (-1.065; 1.023)	-0.003 (-0.097; 0.092)	0.952	0.088 (-0.014; 0.184)	0.102
Carotid intima-media thickness† (cIMT) (n=726)					
Model 2 + MSG_{CGM} (λ=0)	-0.871 (-35.641; 31.033)	-0.003 (-0.128; 0.109)	0.954	0.080 (-0.038; 0.201)	0.196
Halfway (λ=0.04)	-0.215 (-34.456; 32.768)	-0.001 (-0.128; 0.120)	0.986	0.075 (-0.045; 0.204)	0.206
Model 2 (λ=0.12)	-1.872 (-32.526; 30.975)	-0.007 (-0.123; 0.111)	0.916	0.078 (-0.038; 0.207)	0.198
Ankle-brachial index† (ABI) (n=816)					
Model 2 + MSG_{CGM} (λ=0)	-0.017 (-0.047; 0.007)	-0.086 (-0.232; 0.038)	0.184	0.042 (-0.057; 0.147)	0.438
Halfway (λ=0.04)	-0.009 (-0.021; 0.001)	-0.045 (-0.105; 0.007)	0.104	0.003 (-0.040; 0.042)	0.898
Model 2 (λ=0.11)	-0.006 (-0.014; 0.000)	-0.033 (-0.071; 0.002)	0.060	-0.008 (-0.032; 0.017)	0.548
Mean circumferential wall stress‡ (CWS_{mean}) (n=725)					
Model 2 + MSG_{CGM} (λ=0)	-1.138 (-3.408; 1.304)	-0.061 (-0.181; 0.073)	0.344	0.085 (-0.045; 0.220)	0.186
Halfway (λ=0.04)	-1.131 (-3.278; 1.165)	-0.060 (-0.172; 0.063)	0.312	0.090 (-0.039; 0.202)	0.160
Model 2 (λ=0.12)	-1.077 (-3.067; 1.218)	-0.059 (-0.169; 0.066)	0.318	0.082 (-0.044; 0.204)	0.180
Pulsatile circumferential wall stress (CWS_{puls}) (n=725)*					
Model 2 + MSG_{CGM} (λ=0)	-0.673 (-1.785; 0.707)	-0.057 (-0.150; 0.058)	0.354	0.049 (-0.065; 0.144)	0.420
Halfway (λ=0.04)	-0.594 (-1.790; 0.788)	-0.049 (-0.148; 0.066)	0.350	0.045 (-0.065; 0.146)	0.434
Model 2 (λ=0.12)	-0.532 (-1.702; 0.643)	-0.045 (-0.145; 0.053)	0.374	0.042 (-0.055; 0.138)	0.410

Regression coefficients (B) indicate the median difference (95% confidence interval) associated with 1 unit (mmol/L) increase of SD_{CGM}. Standardized regression coefficients (st.β) indicate the median difference (95% confidence interval) associated with 1 SD higher SD_{CGM} or MSG_{CGM}. All coefficients were estimated by use of ridge regression. We pragmatically chose the level of penalization based on the λ required to reduce the variance inflation factor (VIF) of Model 2 + MSG_{CGM} back to the VIF of Model 2 (or halfway back). Point estimates and 95% confidence intervals were calculated by use of 1,000 bootstrap estimates.

The associations were adjusted for age, sex, educational level, mean arterial pressure (*), office systolic blood pressure (†) or brachial pulse pressure (‡), heart rate (in case of cf-PWV and ABI only), body mass index, smoking status, alcohol use, total-to-HDL cholesterol levels, use of antihypertensive and lipid-modifying drugs, and the other CGM-assessed index.

ESM Figure 1. Flowchart delineating the derivation of the study populations

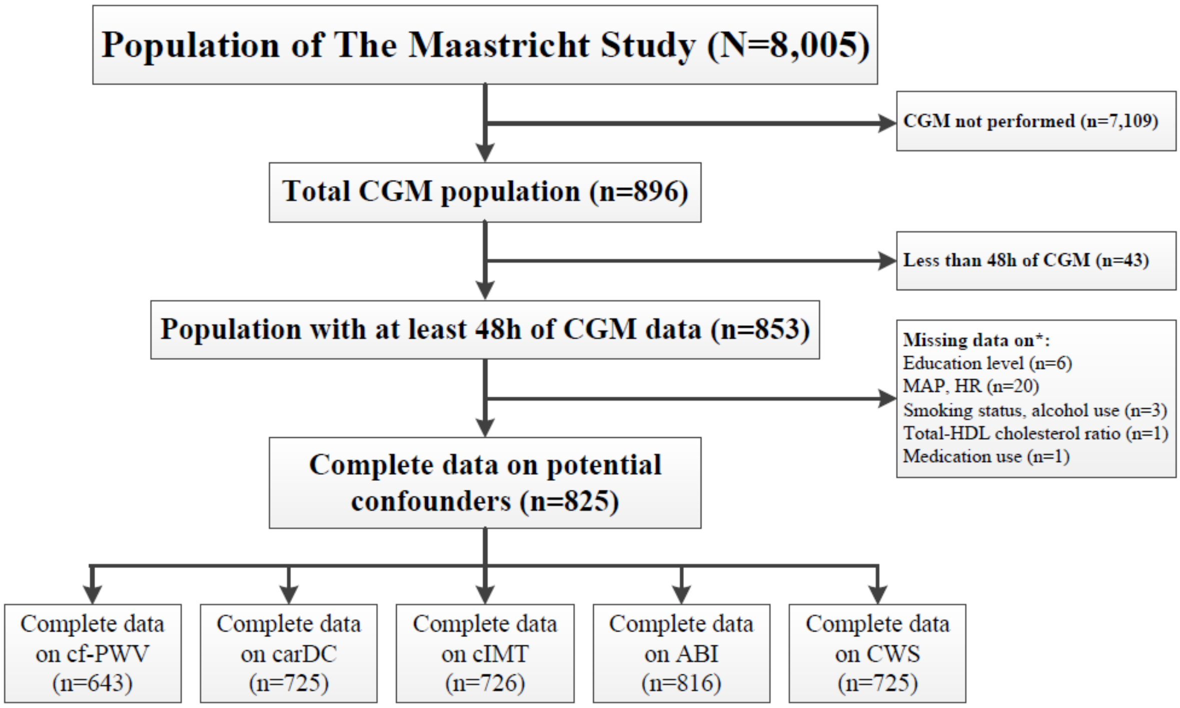

CGM, continuous glucose monitoring; MAP, mean arterial pressure; HR, heart rate; HDL, high-density lipoprotein; cf-PWV, carotid-femoral pulse wave velocity; carDC, carotid distensibility coefficient; cIMT, carotid intima-media thickness; ABI, ankle-brachial index; CWS, circumferential wall stress.

* Not mutually exclusive.

ESM Figure 2. GMS-highlighted scatter plots of the associations of CGM-assessed SD (SD_{CGM}) with carotid-femoral pulse wave velocity and carotid distensibility coefficient

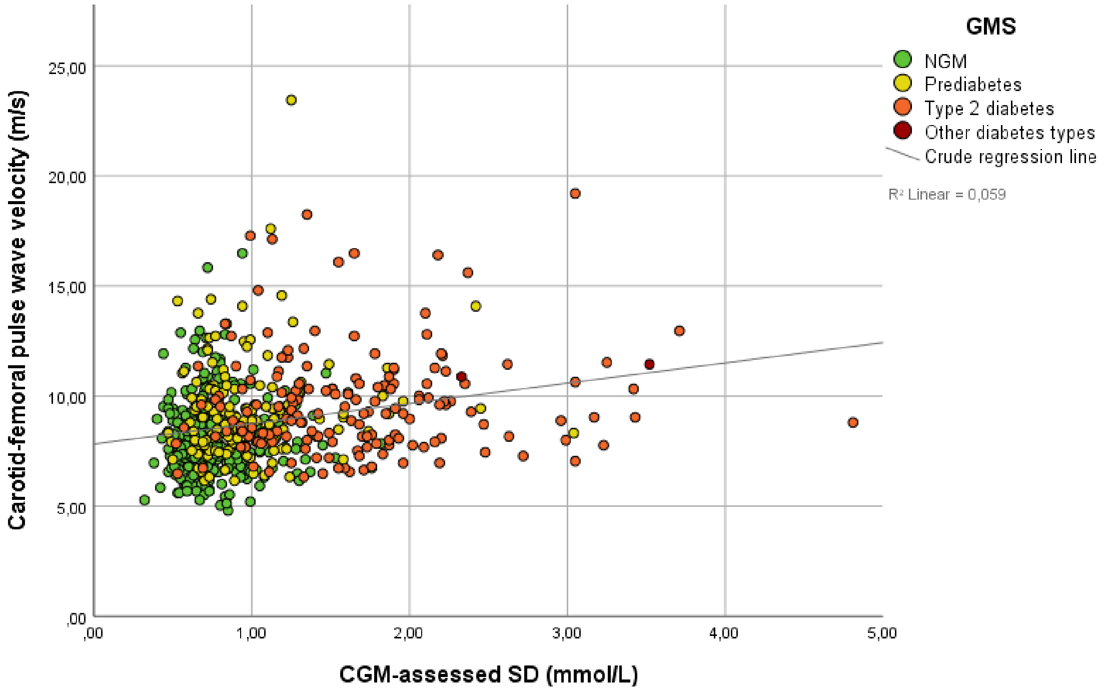

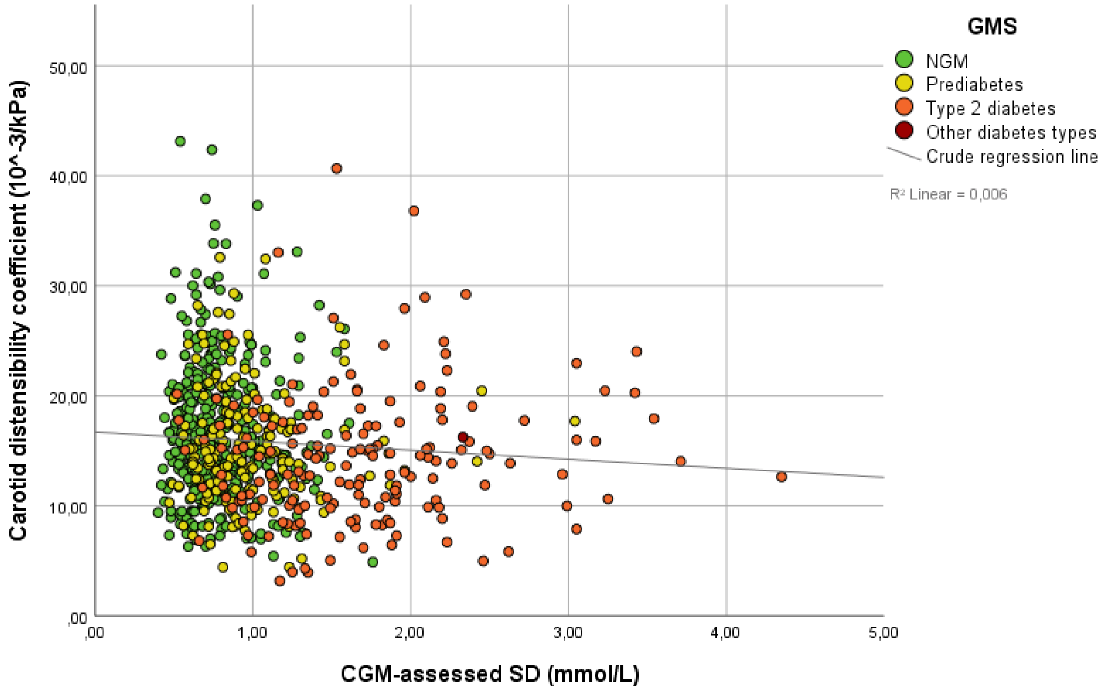

CGM, continuous glucose monitoring; SD, standard deviation; GMS, glucose metabolism status; NGM, normal glucose metabolism.

ESM Figure 3. GMS-highlighted scatter plots of the associations of CGM-assessed SD (SD_{CGM}) with carotid intima-media thickness and ankle-brachial index

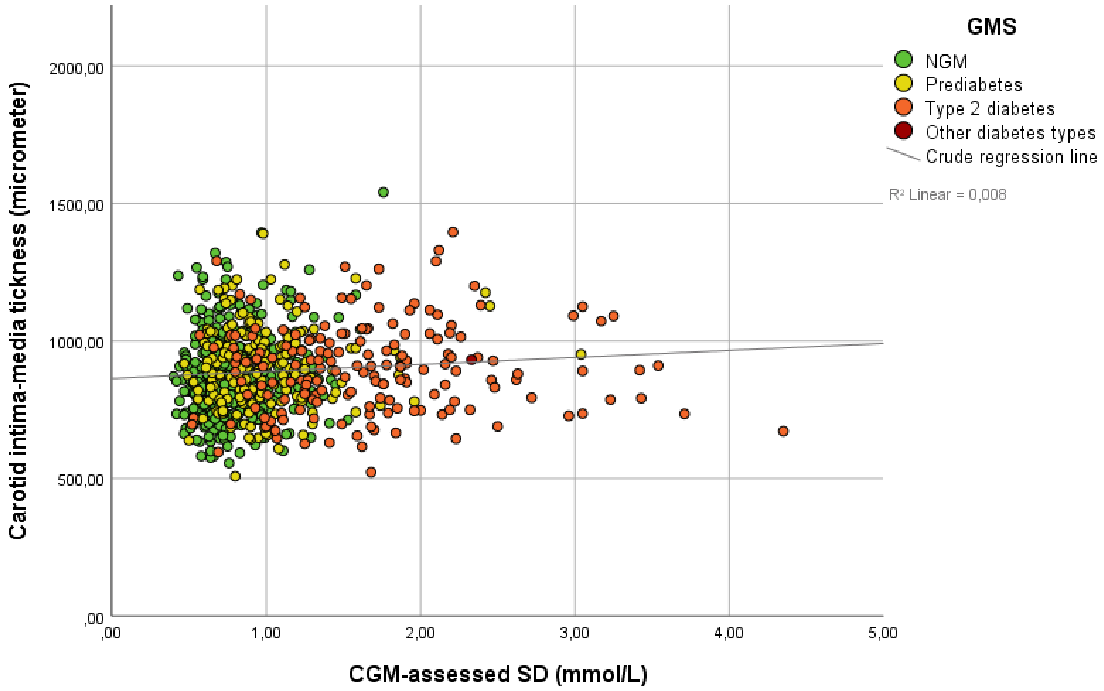

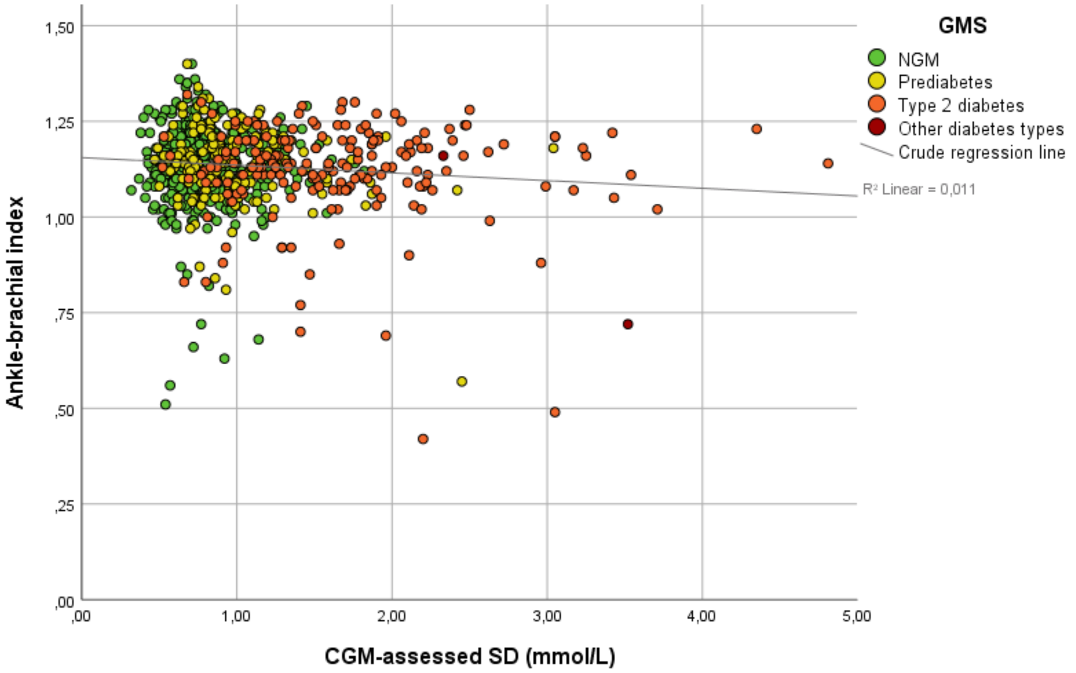

CGM, continuous glucose monitoring; SD, standard deviation; GMS, glucose metabolism status; NGM, normal glucose metabolism.

ESM Figure 4. GMS-highlighted scatter plots of the associations of CGM-assessed SD (SD_{CGM}) with mean and pulsatile circumferential wall stress

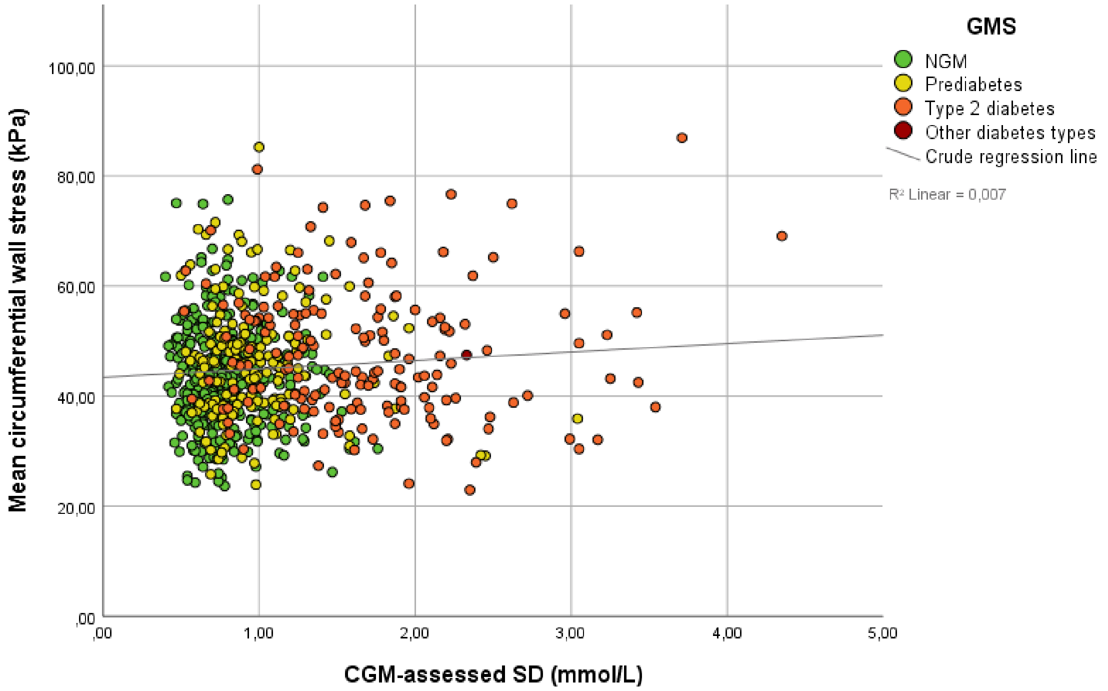

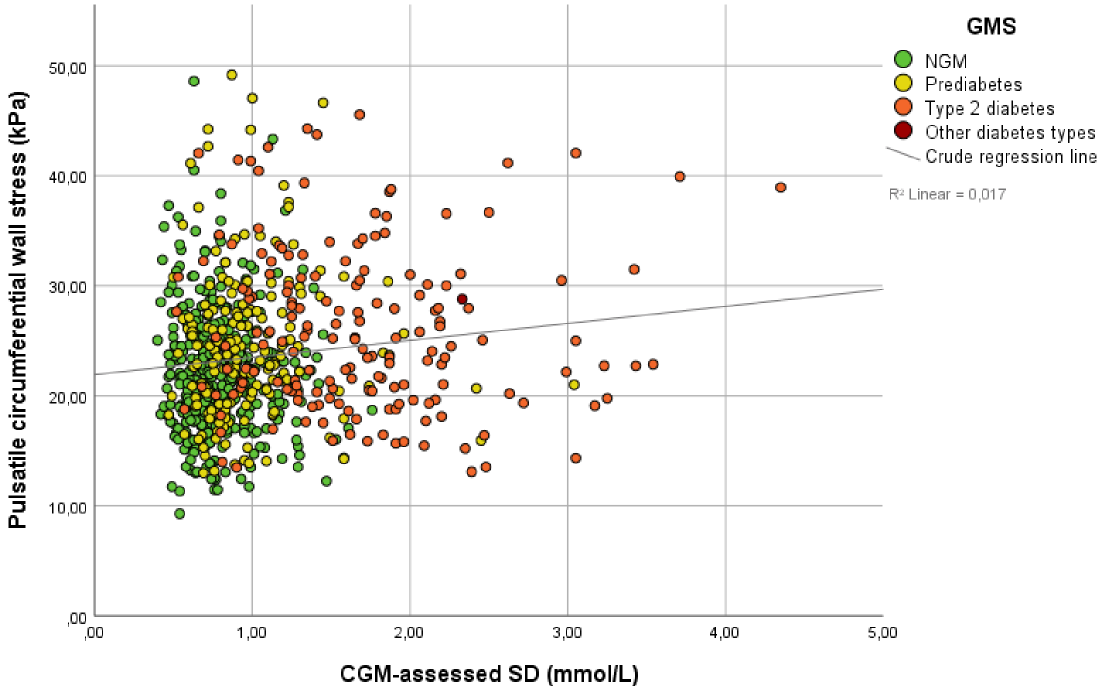

CGM, continuous glucose monitoring; SD, standard deviation; GMS, glucose metabolism status; NGM, normal glucose metabolism.
